# The identification, logic and enlightenments of intra-urban place communities in China

**DOI:** 10.1038/s41598-021-03917-1

**Published:** 2022-01-07

**Authors:** Xie Yang, Zhang Jie, Chen Xiao

**Affiliations:** 1 Beijing Tsinghua Tongheng Urban Planning & Design Institute, Beijing, 100085 China; 2 Tsinghua University, Haidian District, Beijing, 100084 China; 3 Beijing University of Civil Engineering and Architecture, Beijing, 100044 China; 4 Beijing Municipal Institute of City Planning & Design, Beijing, 100045 China

**Keywords:** Environmental economics, Environmental social sciences, Socioeconomic scenarios

## Abstract

Spatial agglomeration phenomena on the earth permeate in various fields of the natural and human world, yet their researches in human society are relatively few with the focus mainly on the economic concept of “industrial clusters”. Precise quantitative descriptions, in-depth logical analyses and proper application approaches for urban planning are lacked in various intra-urban spatial agglomeration phenomena. By using over 10 million POIs in the mainland China, 18 grid network models with two varieties of spatial relationships (co-location/adjacent) are constructed in this article. 23 typical place communities are extracted based on complex network analysis, and four types of agglomeration driving forces are summarized. A comprehensive demonstration displaying the application process of co-location/adjacent place matrices in auxiliary decision of the implanted place types is carried out with the example of the revitalization project of Taoxichuan Area in the city of Jingdezhen.

## Introduction

The phenomenon of spatial agglomeration essentially caused by the uneven occupancy of the earth’s surface is prevalent in various systems of natural and human world^1,2^. Major studies are conducted in the field of ecology with the relevant concepts such as cluster (agglomeration of the same species) and community (agglomeration of various species) being proposed to describe the spatial phenomenon of species distribution^3–5^. In human world, similar agglomeration phenomenon of man-made “concrete forests”—the various types of buildings built by human beings as containers for specific types of production and living behaviors, deserves the same in-depth study as ecological agglomeration phenomenon.

The earliest Chicago school of sociologists proposed monocentric and polycentric models of urban spatial structure^6^, zoning cities by functional and socio-economic attributes. And the concept “occupancy of the earth's surface” was put forward by Geographer Allen K. Philbrick to construct a multi-scale spatial unit system formed by the artificial built environment occupying the earth^1^. Later, relevant researches mainly transferred to the field of economics with a higher priority on the study concerning microscopic mechanism of agglomeration phenomenon. Marshall first discussed the phenomenon of industrial agglomeration on the topic^7^, and then Arrow and Romer further elaborated and developed it into the Marshall-Arrow-Romer (MAR) theory of spillover effects^8,9^ which emphasized the knowledge spillover within the industrial cluster. Jacobs attached much importance to the role of urban environment on knowledge spillover and innovation incentives^10^, which was called the Jacobs spillover effect, emphasized more on knowledge spillover from inter-industry partnerships. Duranton systematically summarized three mechanisms of micro agglomeration—sharing, matching and learning^11^. Following the concept of "industrial cluster" being first explicitly raised by Porter^12^, extensive researches on industrial clusters were conducted by using a large number of quantitative measures, such as the Herfindahl index^13,14^, the locational Gini coefficient^15,16^ and the Hoover localization coefficient^17,18^ to quantify industrial clusters within a sector; and other quantitative papers measured industrial clusters among different sectors by co-agglomeration or co-location indices^19–22^.

To sum up, the attention on the spatial agglomeration phenomenon is mainly paid to industrial clusters in the economic field and statistical aggregated data is mostly used at the national scale or inter-city scale, which has two obvious shortcomings. First, oblivious of the agglomeration phenomena caused by factors other than the economy, such as the concentration of administration and public services that are not entirely governed by market laws, are based on political, environmental and historical ties^23^. Second, agglomeration phenomenon at the finer spatial scale within cities^24,25^ has not been given enough attention owing to the lack of fine geospatial data.

As is known to us all, the dense concentration of socio-economic factors and activities, one of the essential characteristics of cities, shapes the urban spatial landscape where different types of places are mixed with each other and distributed unevenly in clusters^11^. Similar to the concepts of “cluster” and “community” in ecology, the “place cluster” concept is defined as a group of places belonging to the same type that gather together within urban built-up areas, and “place community” as a group of places belonging to different types. Since different types of places are always highly mixed in urban space, the study on place communities is of more practical significance. In addition, it’s also vital to study the micro mechanism and scale characteristics of intra-urban place communities for the development of spatial economic theories, micro-scale site selection of enterprises, and especially urban planning and design practice, in which the proper spatial unit is a core issue to construct a more efficient and sustainable urban spatial structure. Place communities may be the ultimate proper spatial units that urban planning pioneers including Howard^26^, Doxiades^27^ and Hall^28^ have been seeking in succession.

This study, inspired from the above weaknesses of the current researches, endeavors to address the following three core questions: (1) How can typical intra-urban place communities be extracted quantitatively? (2) In which route do places inter-relate logically among place communities? (3) What are the practical enlightenments of intra-urban place community research for urban study and planning practice?

**Figure 1:**
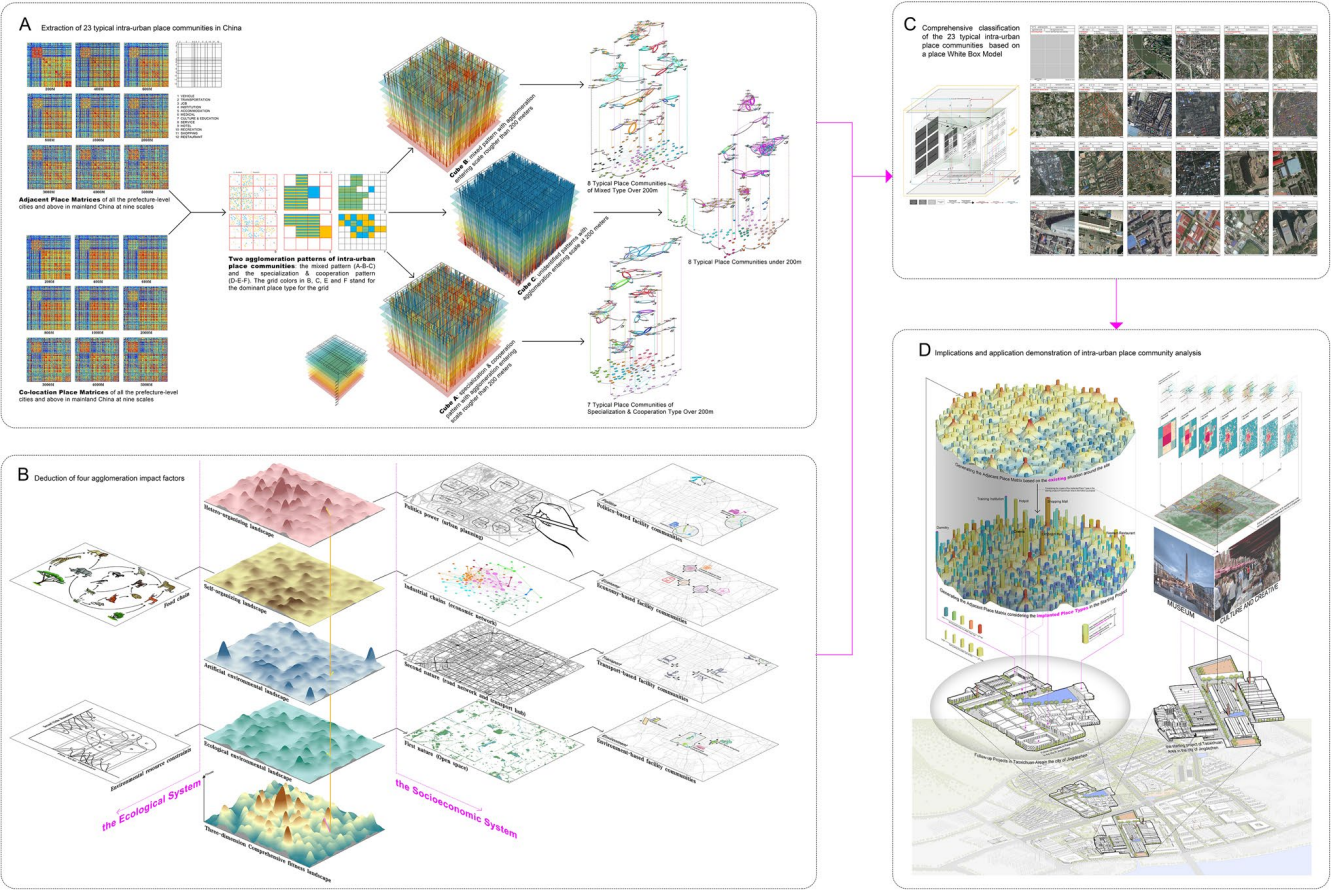
The flow chart showing the key steps of the research.

To address these three core questions, (1) a quantitative data-driven approach is designed to extract 23 typical intra-urban place communities in China (Graph A in Fig. 1). (2) Then Four types of agglomeration forces are deducted (Graph B in Fig. 1), and the 23 typical place communities are classified into the four agglomeration force groups with the aid of a place White Box model (Graph C in Fig. 1). (3) Finally, a comprehensive demonstration displaying the application process of co-location/adjacent place matrices in auxiliary decision of the implanted place types is carried out with the example of the revitalization project of Taoxichuan Area in the city of Jingdezhen, China (Graph D in Fig. 1).

## Results

### Extraction of 23 typical intra-urban place communities in China

Based on our perception of daily life phenomena, there are two spatial patterns of "specialization and cooperation" and "mixed" (Graph A in Fig. 1). This so-called "specialization and cooperation" pattern refers to the place communities in which the same types of places form a place cluster (specialization), and different place clusters locate adjacent to each other (cooperation). For example, similar manufacturing enterprises are distributed in the same industrial park forming a manufacture cluster at a small scale, and a snack cluster composed of many snack shops close to this manufacture cluster at a larger scale. The so-called "mixed" pattern refers to those place communities that are highly mixed within all scales. For instance, cold drink shops and restaurants usually cluster closely together at any scale.

It is of great significance for extracting urban spatial units in urban planning and design practice to distinguish these two patterns. Thus we creatively propose a grid adjacent network analysis method on the basis of grid co-location probability concept in the evolutionary economic geography^29–34^. This concept can be commonly understood as the probability of two place types locating on two adjacent grids. Therefore, the simultaneous analysis of grid co-location probability and grid adjacent probability can distinguish the “specialization and cooperation” from “mixed” patterns among intra-urban place communities. As the grid scale becomes rougher, a pair of place types enters the “agglomeration mode” at a certain scale when both the co-location and adjacent probability of these two place types are considerably high (above the top quartile), and exits from the “agglomeration mode” when either of the co-location and adjacent probability is below the top quartile. Prior to the agglomeration mode, if the pair's co-location probability is significantly high with the exclusion of its adjacent probability, the agglomeration pattern of this point pair is defined as the “specialization & cooperation” pattern; otherwise, it is defined as the “mixed” pattern. In this paper, the pairs that have entered the agglomeration mode twice or more are excluded since they are in an unstable agglomeration state.

As the minimum grid in this study is at 200-m scale, it is impossible to distinguish the mixed pattern from the specialization and cooperation pattern when a place pair enters the agglomeration mode at 200 m. Therefore, we will discuss this situation separately which calls for the following-up studies concerning finer scales than 200 m.

**Figure 2:**
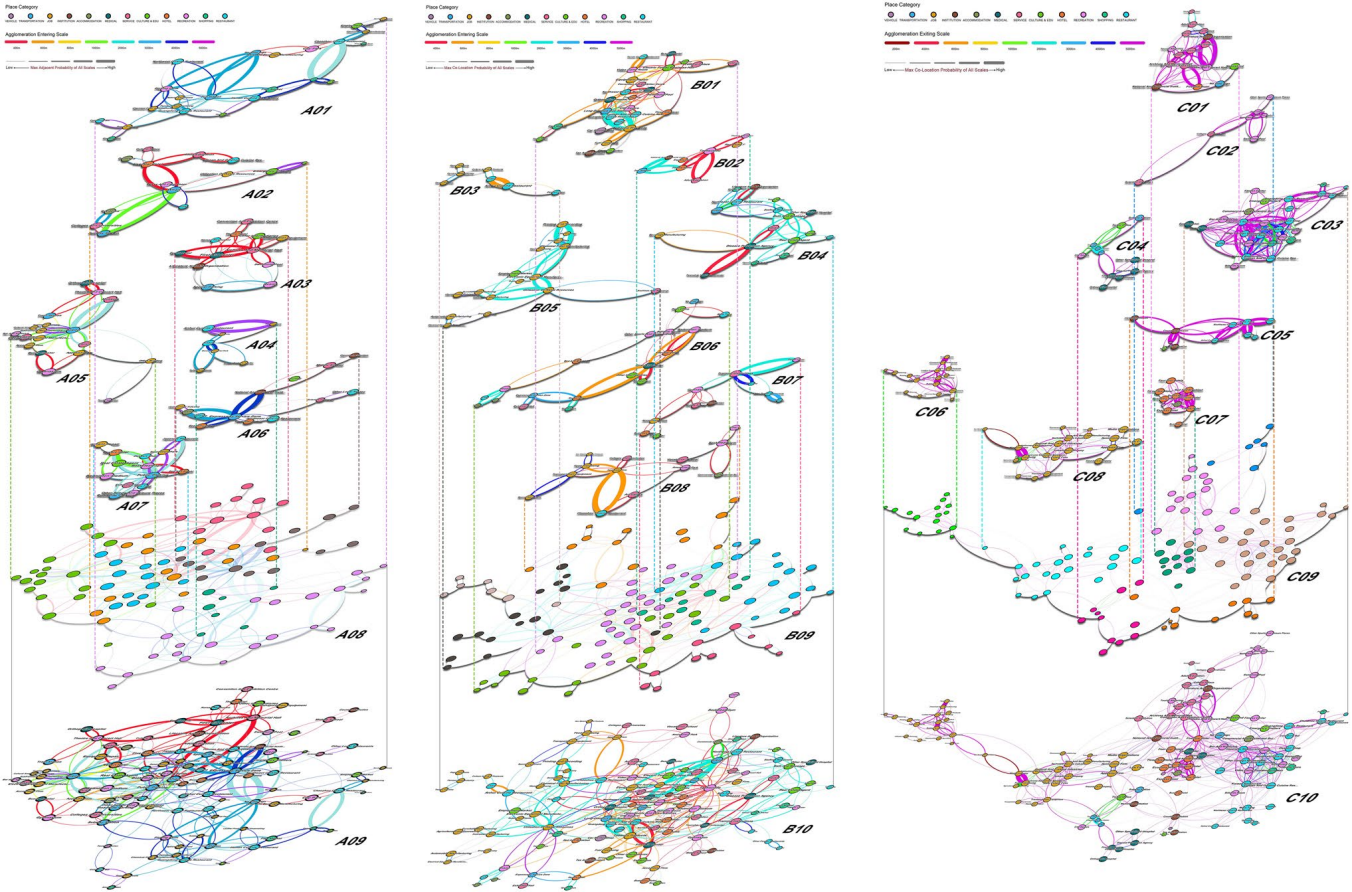
Typical place communities (A01–A07, B01–B08, C01–C08) extracted from the significant agglomeration place network (A09, B10, C10) based on the modularity algorithm in Gephi 9.2 software (Graph A08, B09 and C09 show the modular groups with unique colors) for Cube A, B and C in this figure.

Three agglomeration cubes are constructed to vividly demonstrate the entering and exiting scales for each place pair (Graph A in Fig. 1). With the aid of these three agglomeration cubes, the significant agglomeration place pairs can be discovered and their agglomeration entering scales can be determined. To further exact typical place communities, the co-location (Cube B and C) or adjacent (Cube A) probability for each place pair at the agglomeration entering scale is normalized and recorded as the edge weight, and we take each place type as a node to construct a significant agglomeration place network for each of the three cubes. Then the total 23 typical place communities are further extracted by using the modularity algorithm in Gephi 9.2 software (Fig. 2).

### Four types of agglomeration driven forces

For the 23 typical place communities extracted from the three agglomeration cubes above, what factors lead to the spatial concentration among different place types? It is far from enough to answer this question only from the economic perspective of Jacobs externality. Since the city is an open dissipative system with both self-organization evolution process and hetero-organization process, within which the law of market economy is merely a rule in the self-organization process. In addition, the distribution of intra-urban places is also affected by environmental, political, cultural and other factors. To sort out the complex factors on the intra-urban place communities, we borrow some inspiration from the ecological niche theory which can well explain the agglomeration force of biological communities in the natural world.

**Figure 3:**
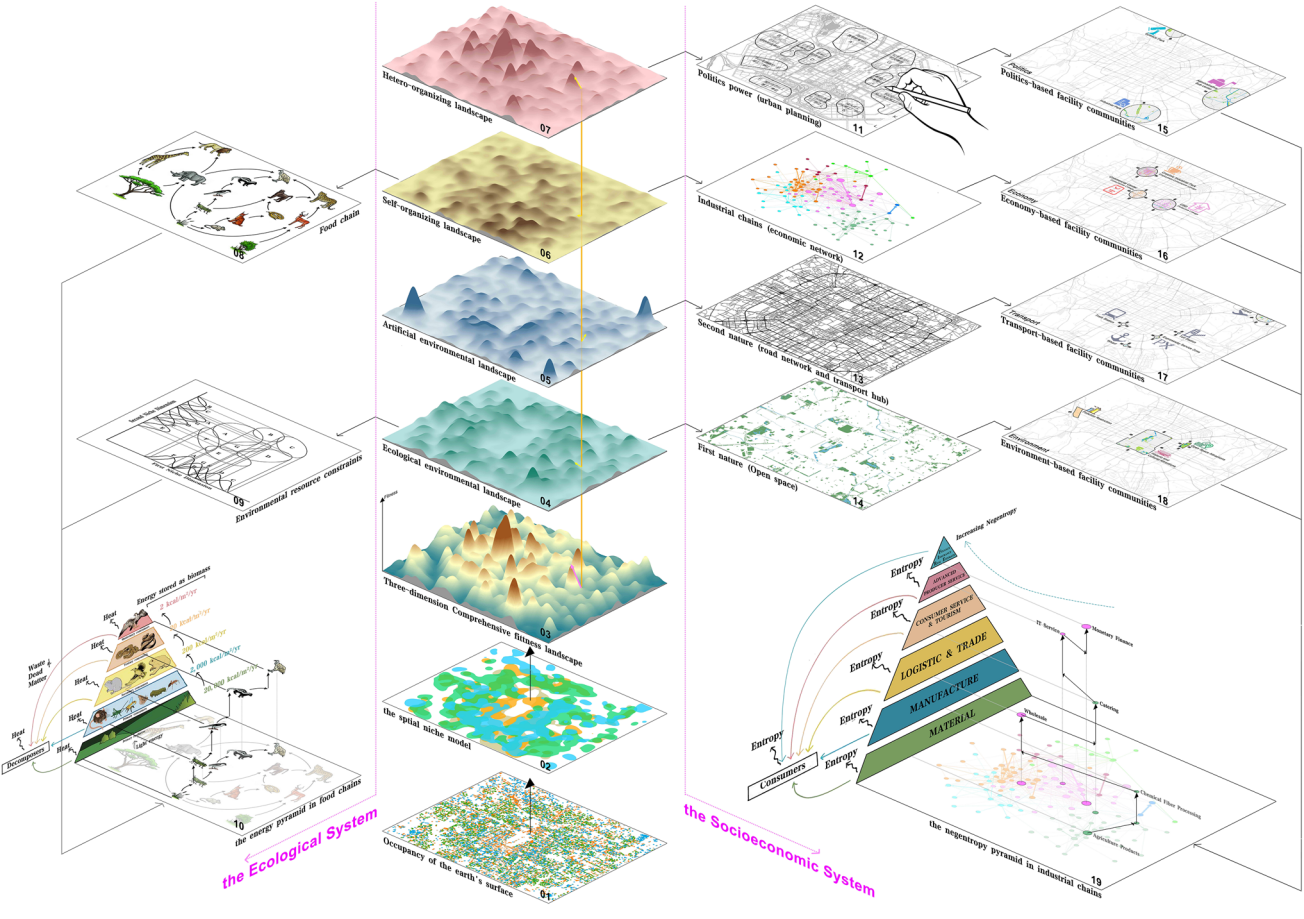
Illustration of key agglomeration driven forces for intra-urban place communities. 01 and 02 express the phenomenon of different types of biological or place species occupying different areas of the earth's surface. 03 illustrates the three-dimensional landscape of the overall attraction function of a location for a species. 04–07 show the four components of the overall attraction function. 08–09 demonstrate the main causes of ecological community agglomeration in ecological niche theory—food chains and species' competition for various natural resources. 10 exhibits the theory of quantified relationships in the study of the intrinsic structure of communities—Lindeman's 10% law of the process of energy flow in the energy-flowing pyramid of food chains. 11–14 exemplify the main causes of the agglomeration of place communities—hetero-organized behavior, self-organized behavior, artificial environment and natural environment. 14–18 elaborates the four types of place communities that correspond to the four main causes—political factor dominated, economic factor dominated, transportation factor dominated and environmental factor dominated. 19 explores a possible approach to the study of the quantitative relationships of the intrinsic structure of the place communities—the pyramid model of negentropic flows in the industrial chains.

The ecological niche theory describes the phenomenon that different species occupy specific habitats and activity areas on the earth through fierce competition for environmental resources and the interaction of complex predation and symbiosis between species^35–37^. Judging from the definition, it is clear to see that there are three core concepts of ecological niche: the first one is the spatial habitat area of species (also generally known as spatial ecological niche, Graph 02 in Fig. 3), the second one refers to the resource constraint set (a high-dimensional space composed of adaptive fitness functions to various environmental resources, Graph 09 in Fig. 3), and the third one is the food chain (predation marks as the most essential interrelationship between species, and there exists Lindeman's 10% law during the process of energy flow through the food chain, forming the biomass pyramid^38^, Graph 08 in Fig. 3).

In light of the ecological niche theory, we proceed to elaborate the agglomeration dynamism within various typical place communities. Here we introduce the concept of overall attraction of a location on the earth’s surface for an ecological/place species (Graph 03 in Fig. 3), which is the sum of the environmental factors and inter-relationships between species that affect the spatial distribution of species. The spatial implication for both ecological and place communities is that a location enjoys high overall attraction for multiple species, which leads to the gathering of multiple species at this location. The overall attraction of a location can be categorized into two components: the environmental component and the inter-specific component. In ecosystems, the environmental component is mainly comprised of abiotic environmental resources such as temperature, soil, and air, and the inter-specific component is mainly linked through the food chain, since predation is the most important relationship among species. Whereas it is more complex for socio-economic place systems with two divergences. First, in addition to purely surface natural attributes, the environmental component also contains the influence of the artificial built environment, which is often referred to as the first nature and the second nature in the discipline of economic geography^39^. The accessibility determined by the structure of the road network directly affects land rent. Just as the fertility of the soil largely determines the total biomass of the ecological community, so the land rent in the socio-economic place system largely determines the intensity of building development. Secondly, the inter-species relationship of socio-economic place system is much more complicated than that in the ecosystem, which can be regarded as a self-organized process governed by the law of evolution^40^. As a mixed self-organized and hetero-organized system, human society boasts the self-organized evolutionary relationship governed by the law of market economy and other centralized hetero-organized behaviors, such as urban planning like various administrative interventions. In summary, there are four components for the overall attraction of location to a place type in socio-economic place system: the natural environment component, the artificial environment component, the self-organized behavior component and the hetero-organized behavior component (Graph 11–14 in Fig. 3). To put it simply, they are environmental, transportation, political and economic factors, which explain the logic of agglomeration dynamism within various typical place communities (Graph 15–18 in Fig. 3).

### Comprehensive classification of the 23 typical intra-urban place communities

**Figure 4:**
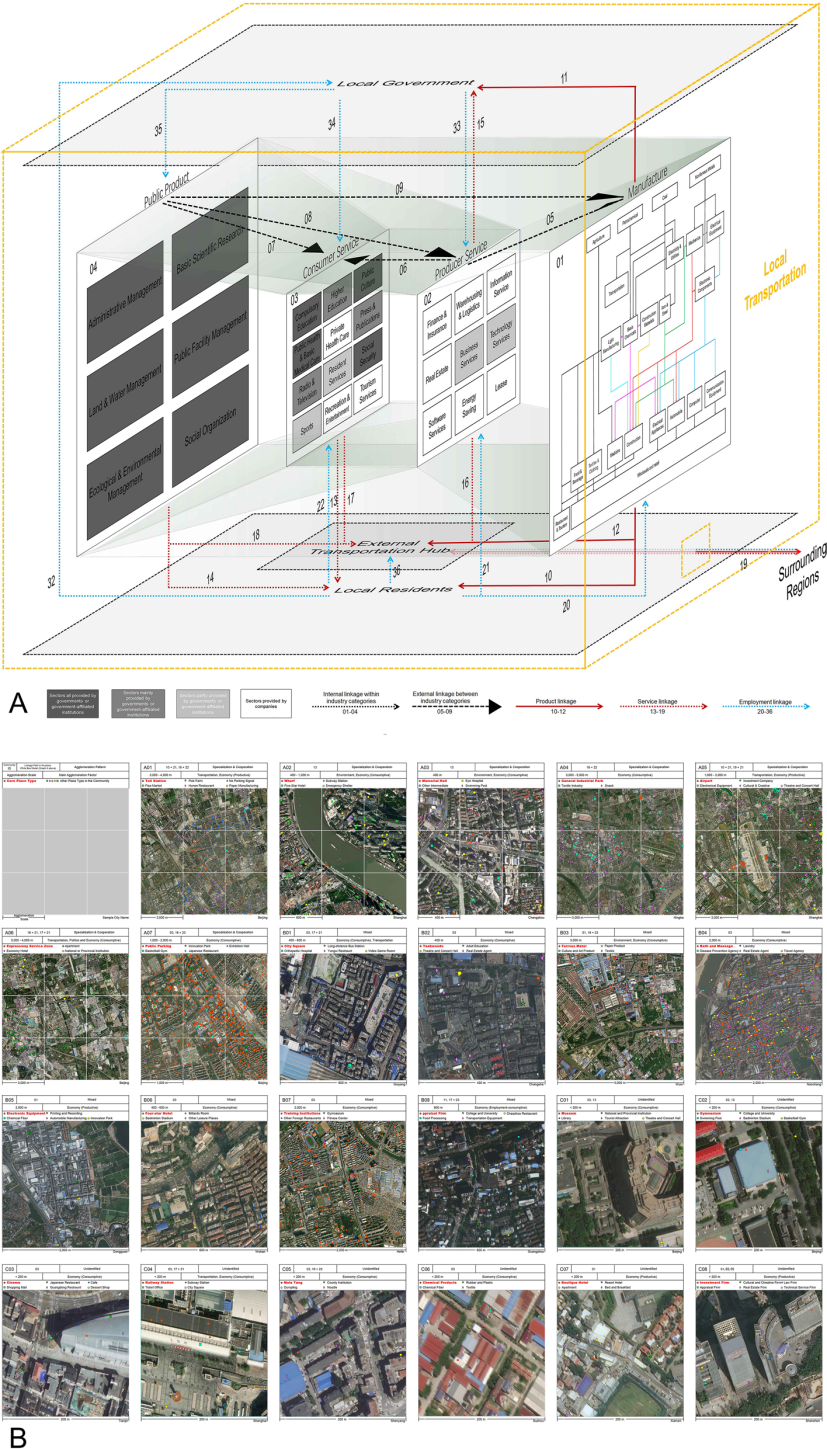
The classification of 23 typical intra-urban place communities by leading agglomeration forces. Graph A illustrates the place white box model—the intra-urban socio-economic process model consisting of various socio-economic subjects based on the input–output table of national economy of China in 2017. Graph B presents the agglomeration force, scale and major place types in each of the 23 typical intra-urban place communities with real-world examples. The satellite maps of the real-world examples were created in ArcMap 10.4 (https://www.esri.com/en-us/arcgis/products/arcgis-desktop/resources), and the base maps were obtained from https://www.arcgis.com/apps/mapviewer/index.html?layers=10df2279f9684e4a9f6a7f08febac2a9.

To examine the intrinsic logic of the intra-urban place communities in a better way, a process model is built to show the specific interactions among the intra-urban places. Based on the input–output table of national economy of China in 2017, we create the “place white box” model (Graph A in Fig. 4)—an intra-urban socio-economic process model consisted of various socio-economic subjects corresponding to different place types. Based on our daily experience, we classify socio-economic subjects into three categories of governments, enterprises and residents, among which the enterprises are categorized into manufacturing, consumer services and producer services, adding public goods provided by the government and various external transportation hubs in an attempt to comprehensively demonstrate the complex interaction dynamism within urban socio-economic systems. The interaction between various socio-economic subjects is realized through various material and information flow.

For the above 23 typical types of intra-urban place communities, their main inner-interaction routes are simulated and categorized into the four impact factors (i.e. political, economic, transportation and environmental) in the place white box model. In particular, the economic linkages are relatively more complex: (1) at the production end, it is mainly linked through the direct supply and demand of products and services between different place types within a place community. (2) at the consumption end, the interaction among consumer places is maintained through the complementary relationship between products and services (such as the relationship between cinemas and restaurants in shopping malls, the attraction of shopping malls to consumers is enhanced as both can meet the needs of consumers for various weekend leisure activities), and the gathering of the same type of places to provide consumers with diversified choice dividends. (3) There is another situation where consumption places gather around production places. For example, snack places gather around industrial parks and government agencies, with the aim to provide work meals for the employed people in these workplaces.

The specific analysis is carried out here to elaborate the internal agglomeration logic of 23 typical place communities in three cubes through the place white box model. Some overall characteristics can be found in the following: (1) place communities for Cube A mainly focus on transportation hubs (airports, highway service areas, toll stations, public parking lots, terminals. (2) The intra-community linkages of place communities for Cube B are mainly economy-guided, which can be roughly divided into production-end linkages and consumption-end linkages. (3) The distinctive feature of place communities in Cube C is the similarity of the place types within the communities, i.e. basically the communities are made up of a large number of similar place types with three main types of interaction: productive, consumptive and employment-consumptive. Eventually, 11 inner interaction linkage types under four main impact factors are summarized into 23 typical place communities (Graph B in Fig. 4).

#### Place communities dominated by economic factors with agglomeration scale finer than 200 m

*Productive linkages* (1) strong linkages in mixed pattern—direct upstream and downstream linkage of industry chain with agglomeration scale finer than 200 m; (2) weak linkages in specialization and cooperation pattern—indirect linkages in industry chain with agglomeration scale of 2000 m.

*Consumptive linkages* By sharing similar consumers, places within the same categories concentrate at the scale finer than 200 m, while places belonging to different categories measure at the scale of 400 m or 2000 m.

*Employment-consumptive linkages* Consumer services gather around major employment places such as governments at the scale finer than 200 m.

#### Place communities dominated by transportation factors with agglomeration scale over 2000 m

*Outward-oriented industries around external transportation hubs* Outward-oriented high-tech manufacturing (electronic components, etc.) and advanced producer services (investment companies, technical services, etc.) gather around the airport, forming communities with a scope of 2000–5000 m in the specialization & cooperation pattern.

*Consumer services surrounding the external transportation hubs* Consumer service places such as fast hotels and local cuisine gather around highway service areas, forming communities with a scope of 3,000–5,000 m.

*Cultural and commercial facilities around inner-city transportation hubs* e.g. cultural palaces and theaters aggregate around public parking lots, forming communities with a scope of 2,000 m.

#### Place communities dominated by environmental factors with agglomeration scale within 400–1000 m

*Communities around beautiful environment and open space* e.g., leisure and entertainment facilities built around waterfront piers with the gathering scope of 400–1000 m.

*Communities boasting historical and cultural resources* Influenced by the urbanization and generation process, these places are equipped with basic urban functions, like hospitals and schools usually locate at historic center areas, forming a type of place communities around memorial halls converted by historical residences or historical buildings at the scope of 400 m.

*Communities with environmental pollution restrictions* Due to the environmental pollution restrictions, chemical, paper and other heavy polluters are often restricted by urban planning regulations to adjacent suburban locations, forming place communities of heavily-polluting industries with the spatial scope of 3000 m.

#### Place communities dominated by political factors with agglomeration scale over 2000 m

*Political communities providing public goods to relocate urban functions* Government, scientific research institutions and other government-supplied places, which are not oriented by market laws, usually distribute for meeting government’s requirements of relocating non-capital functions. Therefore, these government’s places usually keep in close proximity to expressway service areas in the suburban, forming a suburban place community of 4000-m spatial scope.

*Communities in industrial parks* There are no direct linkages among some manufacturing industries (e.g., agricultural processing and furniture manufacturing), but they concentrate together because of the industrial park development strategy at the scale of 2000 m.

## Discussion

What are the real-world takeaways with the 23 typical intra-urban place communities in China based on co-location/adjacent matrices under four types of agglomeration forces? The answer lies in at least two aspects: one is due to the fact that the intra-urban place community research provides precise quantitative evidences of proper mixture scale of urban land use for urban study; and the other significance is that it can be properly transformed into a set of big-data auxiliary tools, which contribute to the judgement of implanted place types in urban design or urban renewal projects.

**Figure 5:**
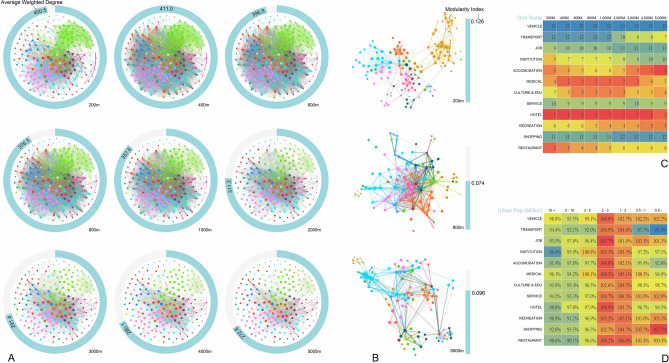
Statistical analysis of co-location place networks of nine scales in China. (**A**) The Average Weighted Degree of the co-location place network within all nine scales. (**B**) The Clustering Index of co-location place network of three scales. (**C**) The Average Weighted Degree rank of each place network at different scales. (**D**) The percentage of the Average Weighted Degree of each place category for different urban population groups while comparing all cities’ samples.

The mixed land use patterns in urban development have been frequently promoted by scholars and governors since New Urbanism, yet there is a lack of solid empirical research concerning the specific co-location mode among all types of urban functions and agglomeration scale. The gap is filled based on our calculation of co-location place matrices (See detail in Supplement Information Note 2-6). (1) the highest mixing degree of the whole place network appears at the scale of 400 m (Graph A in Fig. 5), revealing that an area within five-minute walk is a basic unit of land use mixture, which requires elastic regulations on land use by regulatory detailed planning. (2) the most significant place network clusters at the scale of 200 m, and the clustering index shows the tendency from decline to rise as the grid scale grows substantially (Graph B in Fig. 5), uncovering a scale-dependent clustering pattern of the intra-urban place network: congeneric agglomeration at small scale, highly mixed at meso scale, and separated layout by main place categories (industrial, residential and recreation) at large scale. This clue is conducive to organizing the spatial structure of urban functions in urban design projects. (3) Different place categories have unique agglomeration scales: economy-triggered agglomeration (restaurant, shopping and hotel) takes effect at small scale, while traffic agglomeration and environment-triggered agglomeration (culture & education, medical) takes effect at large scale and meso scale respectively (Graph C in Fig. 5). This conclusion further confirms the former analysis about the agglomeration scale of the 23 typical place communities, and sheds light on the micro-scale site selection of enterprises to integrate into industrial chains and place communities. (4) Urban population scale has a far-reaching impact on mixing pattern among different place categories. To put it in detail, medical places have relatively high mixing degree in large cities due to the intensive supply of medical resources to large cities, basic urban functional places (shopping and job) enjoy higher mixing degree in small cities. And middle-sized cities have the highest mixing degree among nearly all kinds of place categories (Graph D in Fig. 5), indicating that middle-sized cities take the lead in terms of life convenience.

Another enlightenment of intra-urban place community is that the co-location/adjacent place matrix can be used to calculate the probability of a certain place type to be implanted into a site based on its own/surrounding existing dominant place types in the practical urban planning and design projects. Here we demonstrate how the co-location/adjacent place matrix can be used in an urban renewal project of Taoxichuan Area in the city of Jingdezhen, China.

**Figure 6:**
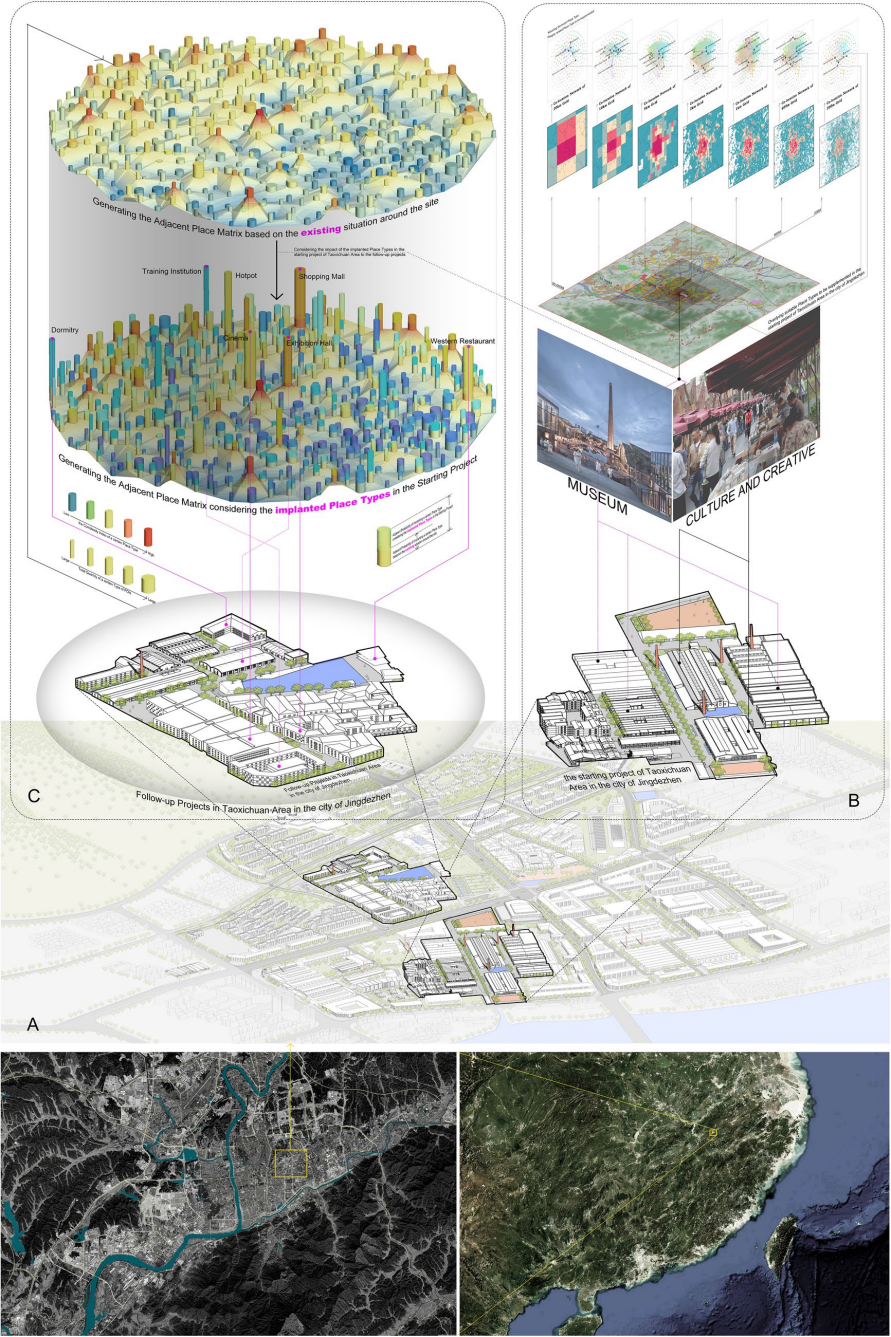
Graphic demonstration of the big-data auxiliary tool based on co-location/adjacent place matrices for the judgement of implanted place types in Taoxichuan Area in the city of Jingdezhen, China. (**A**) The site place of Taoxichuan Area. (**B**) The illustration of co-location matrix for the start-up Porcelain Factory plot. (**C**) The illustration of adjacent matrix for the follow-up Wannengda Porcelain Factory plot. The satellite maps in Graph A were created in ArcMap 10.4 (https://www.esri.com/en-us/arcgis/products/arcgis-desktop/resources), and the base maps were obtained from https://www.arcgis.com/apps/mapviewer/index.html?layers=10df2279f9684e4a9f6a7f08febac2a9.

Jingdezhen, located in the northeast of Jiangxi province, is the world-renowned ceramic capital and honored as “the home of ceramics” for thousands of years in China. Between the 1980s and 1990s, with the implementation of China's reform and opening-up policy, state-owned ceramic factories went bankrupt, abandoned and declined, leaving large areas of industrial heritage, among which Taoxichuan area composing of five state-owned ceramic factories is the largest with an area of 1.2 km^2^ (Graph A in Fig. 6). For the judgement of implanted place types in the transformation and upgrading of such a large area, the application of co-location/adjacent place matrix is divided into the following two types.

1. One is the start-up plot of the whole area—Porcelain Factory plot (the plot with clear property rights and the best infrastructure in the whole area), which mainly adopts the co-location place matrix method (Graph B in Fig. 6). The spatial grids of different scales within the plot are being divided, and then the remaining dominant place types at each scale is being extracted to figure out the place types with the highest weighted sum of co-location probability (i.e. the place types that meet the needs of the existing socio-economic activities and people). It is found that cultural and creative industry and museum are the most suitable place types supported by the existing place types of all scales. These two place types have been constructed and operated as the dominant place types to guide the revival and regeneration of the Cosmic Porcelain Factory plot.
2. The second is other follow-up plots except the start-up one. Once the porcelain factory has been completed as the start-up plot, it is of great significant to foster appropriate place communities surrounding those already implanted in the start-up plot to form sound functional interaction. Therefore, the adjacent place matrix comes to handy for the judgement of implanted place types in the follow-up plots. Taking Wannengda Porcelain Factory area as an example, ① at first, we just consider the existing dominant place types before the completion of the start-up plot, and calculate the weighted sum of co-location probability with all the existing dominant place types, thus generate the suitable forest map of implanted place type meeting the surrounding functional needs of Wanengda plot (the height of each platform on the forest base represents the complexity of the business type; each column represents a place type with the height of the column referring to the implantation suitability calculated based on the adjacent probability matrix and the thickness indicating the total amount of POIs belonging to this place type, and the color presenting the complexity of the place type). (2) Then, various place types implanted in the start-up plot are also added to the summation calculation of adjacent probability, showing the great impact of the completed cosmic porcelain factory on the surrounding plots. Since the supporting place types of culture and creative industry such as training institutions, exhibition halls, cinemas, shopping centers and supermarkets enjoy high suitability, they are well arranged in the Wannengda plot to fit the start-up porcelain factory, fostering a suitable place community around cultural and creative industry.

This study adopts a data-driven approach to explore the scale and logic of typical intra-urban place communities in Chinese cities, which is of pioneering significance. But several aspects still exist to be further developed. First, analysis of finer gird scale than 200 m can be carried out to unlock the unsolved scale mystery of many places gathering at a more micro level, such as similar shopping places in the same category. Second, the quantitative calculation methods of various places in the place community can be further explored by constructing the negentropy flow pyramid of industry chains in the place niche theory corresponding to the energy flow pyramid of food chain in the ecological niche theory. Finally, the following question can be taken into consideration in the follow-up research: what is the overall layout structure of the place communities within the city? This is a crucial issue conductive to put the pieces of place communities together into a complete puzzle of the whole urban area.

## Methods

Drawing on a series of measures of industry co-location probability from the literature on complex economics^41–43^, the co-location/adjacent place networks at different scales are constructed by the following steps.

### Classifying POI

POI (Point of Interests) serves as a good representation of intra-urban places. The data used here are extracted from Gaode Map POI within the built-up areas (2018 built-up area boundaries in GHSL database^44^) of cities at the prefecture level and above in the mainland China in 2018. Based on the original classification, with reference to the National Economic Industry Classification (GB/T-4754-2017) and the Statistical Classification of Living Services (2019), 12 categories and 210 types of POI classification finally form in the spirit of minimizing gaps in the number of various POI types by further subdividing those POIs in large quantities, and combining the ones in small numbers (See detail in Supplement Information Note 1).

### Dividing urban built-up areas into grids

In order to explore the significant agglomeration scales of intra-urban place clusters and communities, we construct a total of 9 spatial grids ranging from 200 m, 400 m, 600 m, 800 m, 1 km, 2 km, 3 km, 4 km to 5 km gradually. It is of little significance to calculate the inter-relationship between two types of places by grids finer than 200 m due to its large quantities and simple form. In addition, there is no need to further scale up since a 5 km grid covers the built-up area of a small city in China. Thus spatial grids containing only one POI are excluded in calculating inter-relation place probability.

### Defining dominant places for a grid

Taking the concept of location quotient (*LQ*) in industrial economics as the reference, this paper defines the *LQ* of each type of places on a grid as the ratio of the number of POIs of a certain type on the grid to the total number of POIs on the grid, divided by the ratio of the number of POIs of that type on all grids to the total number of POIs on all grids, which is shown below in the calculation formula (1) with ${x}_{i}^{\left(m\right)}$ and ${LQ}_{i}^{(m)}$ referring to the number and locality degree of POIs of Place *i* in grid *m* respectively. Place *i* dominates in grid *m* when the *LQ* is greater than 1.

1 $${LQ}_{i}^{(m)}=\frac{({x}_{i}^{\left(m\right)}/{\sum }_{i}{x}_{i}^{\left(m\right)})}{({\sum }_{m}{x}_{i}^{\left(m\right)}/{\sum }_{m}{\sum }_{i}{x}_{i}^{\left(m\right)})}$$

Then the place universality *U*_*i*_^*M*^ is defined as the number of grids in which Place *i* appears as the dominant place (${LQ}_{i}^{(m)}>1$) for a set *M* of grids at any scale. Then *U*_*ij*_^*M*^ is the number of grids in which a certain two types of Place *i* and Place *j*, appearing together as dominant places (${LQ}_{i}^{(m)}>1$ and ${LQ}_{j}^{(m)}>1$).

### Constructing the co-location place matrix

The co-location probability ζ between two types of places is calculated based on the concept of conditional probability. *N* is the total number of grids in the set *M*.

2 $${\zeta }_{ij}=\frac{{U}_{ij}^{M}*N}{{U}_{i}^{M}{*U}_{j}^{M}}$$

In short, the co-location probability between two types of places means the probability of two types of places co-existing on the same grid as the dominant places. The minimum value is 0. It equals 1 if the two types of places are completely independently distributed spatially; and a larger value means the greater probability that the two types of places are co-located, the more likely they are to appear together as dominant places on the same grid.

It is worth noting that the co-location probabilities need to be normalized across scales before a cross-scale comparative study can be conducted while determining the most significant co-location agglomeration scale for a place pair.

### Constructing the adjacent place matrix

The co-location place network reveals the co-location probability of places within a certain spatial grid, but fails to show the inter-relationship of places between grids due to the lack of consideration on the overall relationship of urban industry structure layout^40^. For this reason, we propose the concept of adjacent place probability to reveal the inter-relationship of places between grids: the probability of the two dominant places’ appearance on two adjacent grids. When it comes to the adjacency relationship, a variety of spatial conceptualization models can be utilized to define it. To make it simpler, all grids with shared boundaries or nodes are defined as adjacent grids in this study (i.e., a typical grid has eight adjacent grids around it, forming a nine-box grid pattern).

The calculation of adjacent place probability is substantially similar to co-location place probability. For a set *M* of grids at any scale, two spatially adjacent grids form a directed grid pair *s*_*pq*_, in which *p* is the source gird meanwhile *q* is the target grid. For two types of places *i* and *j*, we define: (1) *U’(p)*_*i*_^*M*^ as the number of grid pairs in which Place *i* appears as the dominant place in the source grid; (2) *U’(q)*_*j*_^*M*^ as the number of grid pairs in which Place *j* appears as the dominant place in the target grid; (3) *U’(pq)*_*ij*_^*M*^ as the number of grid pairs in which Place *i* appears as the dominant place in the source grid and Place *j* appears as the dominant place in the target grid at the same time; (4) *N’* is the total number of grid pairs in the set *M*. Thus the adjacent probability of Place *i* and *j* in the grid set *M* at any scale can be defined as:

3 $${\zeta {^{\prime}}}_{ij}=\frac{{U{^{\prime}}(pq)}_{ij}^{M}*N {^{\prime}}}{{U{^{\prime}}(p)}_{i}^{M}*{U{^{\prime}}(q)}_{j}^{M}}$$

In this way, the co-location place network matrix and the adjacent place network matrix at nine scales can be constructed for all prefecture level cities and above in the mainland China (Graph A in Fig. 1) (Supplementary Notes).

## Supplementary Information

Supplementary Information 1.

Supplementary Information 2.

Supplementary Information 3.

## Data Availability

All data that we use in this study is publicly available. The POI data was obtained through Gaode Map API in 2018, https://lbs.amap.com/. Built-up area data in 2018 was obtained from GHSL database, https://ghsl.jrc.ec.europa.eu/download.php. The spatial grid data with POI numbers at 200-m scale generated in this work is provided in supplementary material.

## Code availability

The analysis in this study was done using ArcGIS and MS Excel. The network layout was generated using Gephi (https://gephi.org/).
