# Supplementary material for: The identification, logic and enlightenments of intra-urban place communities in China
Source: Sci Rep. 2022 Jan 7;12:247. doi: 10.1038/s41598-021-03917-1 (PMC8741909; doi:10.1038/s41598-021-03917-1)
Supplement: Supplementary file 1 — Supplementary Information 1. [file 41598_2021_3917_MOESM1_ESM.docx]

Supplementary information for

The Scale, Logic and Structure of Intra-urban Place Communities Based on Place Niche Theory

**Supplement Notes**

**Supplement Note 1: Classification of POI 3**

**Supplement Note 2: Global characteristics of place networks 5**

**Supplement Note 3:** **Concentration scale heterogeneity of 12 place categories 7**

**Supplement Note 4: Concentration scale heterogeneity of 210 place types 10**

**Supplement Note 5: Network modularity of 210 place types 13**

**Supplement Note 6: Concentration scale of typical place clusters 24**

**Supplement Note 1: Classification of POI**

**Supplement Figure 1** The number of all categories and types of POIs (Point of Interest) in this research.


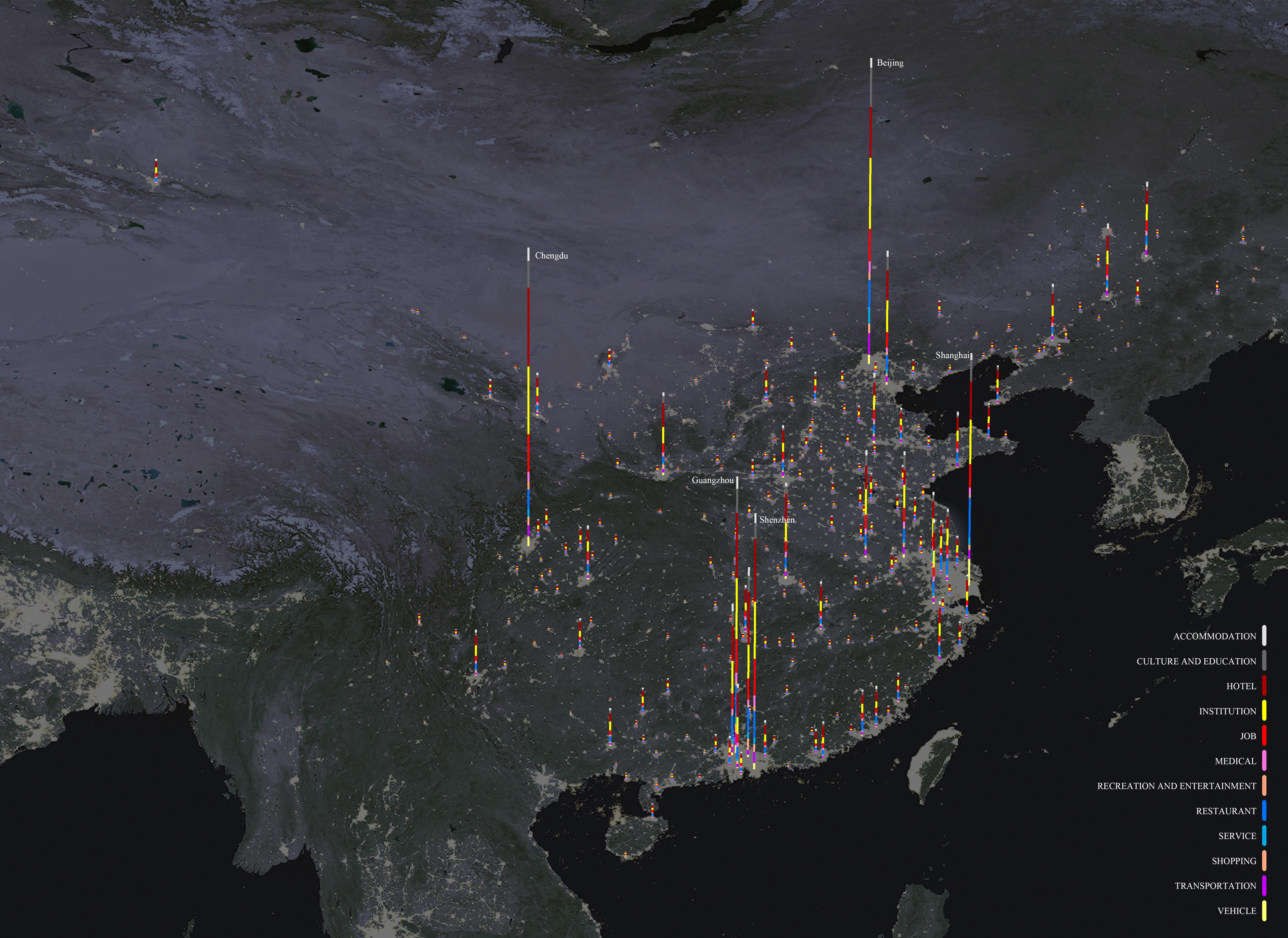


**Supplement Figure 2** The number of twelve categories of POIs for 276 prefecture-level cities and above in the mainland China, created in ArcScene 10.4 (<https://www.esri.com/en-us/arcgis/products/arcgis-desktop/resources>). Each column represents a city, and the column height stands for the total number of POIs in this city. Some cities are excluded due to poor data quality, and spatial grids with only one POI are abandoned. The night light map was obtained from <https://earthobservatory.nasa.gov/features/NightLights>.

**Supplement Note 2: Global characteristics of place networks**


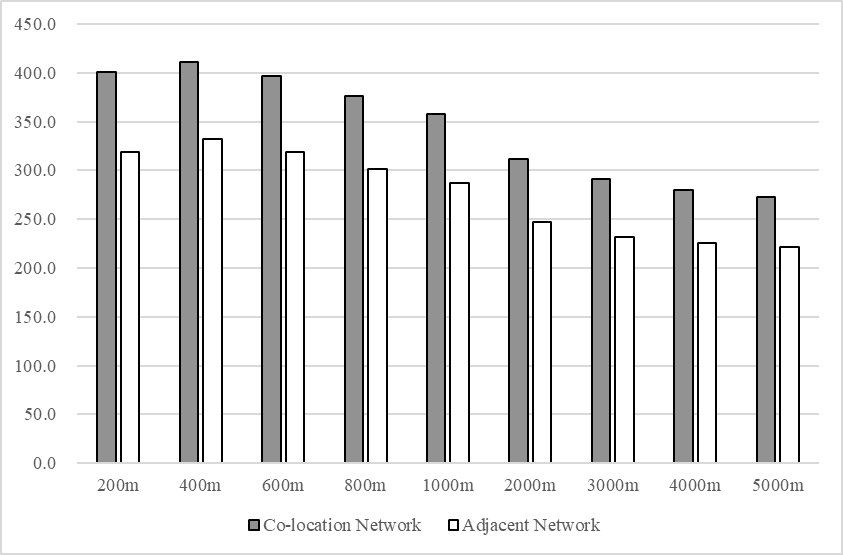


**Supplement Figure 3** Average Weighted Degree of each place network at different scales. It shows that places at 400-meter scale have the densest interrelationship for both the co-location and adjacent network.


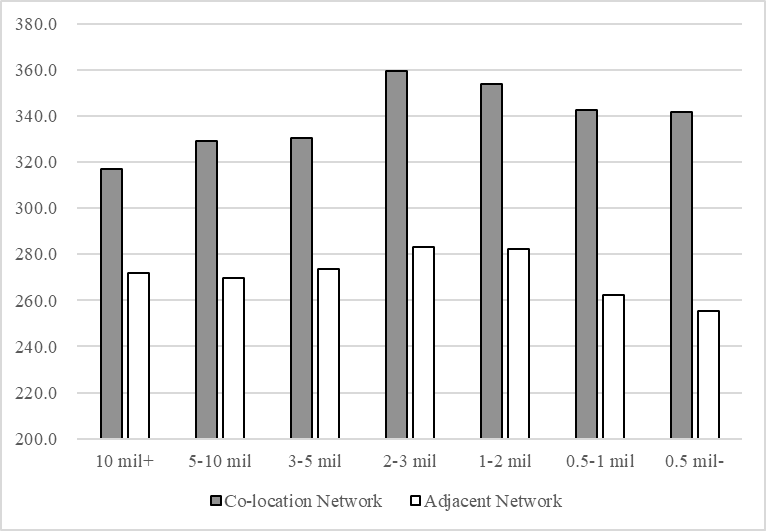


**Supplement Figure 4** The average value of Average Weighted Degree at all nine scales for different urban population groups. It shows that large cities with 1-3 million population have the highest place mixing degree, and mega-cities with over 300 million population have sparse co-location\adjacent relationship. Urban population data is obtained from *Statistical yearbook of urban construction in China (2017).*

**Supplement Figure 5** Average Weighted Degree of each place network at different scales for different urban population groups. The highest place mixing degree for mega-cities with over 10 mil population appears at 600-meter scale, and at 400-meter scale for other cities.

**Supplement Note 3: Concentration scale heterogeneity of 12 place categories**

**Supplement Figure 6** Average Weighted Degree of each place network at different scales for different place categories. All categories except job have the highest value at the scale of 400 meters.

**Supplement Figure 7** Average Weighted Degree rank of each place network at different scales. The highest rank for restaurant, hotel, shopping, institution and job appears at the small scale, for culture & education and hospital it appears at the meso scale, and for accommodation, transportation and recreation it appears at the macro scale.

**Supplement Figure 8** The percentage of the Average Weighted Degree of each place category for different urban population groups compared with the sample of all cities. It shows that each place category has high mixing degree in middle-size cities with 1-3 million population. In addition, Average Weighted Degree of shopping, vehicle is relatively high in small cities, and Average Weighted Degree of hospital is relatively high in large cities

**Supplement Figure 9** The percentage of the Average Weighted Degree of each place category at different spatial scales for different urban population groups compared with the sample of all cities. The table of total cities shows the value of Average Weighted Degree for all sample cities, and the other tables represent the percentage of a population group compared with the total sample. Compared with the average level of all cities, the mixing degree of various place categories in small cities is higher at a large scale, the mixed degree of medium-sized cities is higher at the meso scale, and big cities have high values at a small scale and low values at a large scale, since the types of places in big cities are more complete meanwhile functional zoning phenomenon is obvious at a large scale in large cities.

**Supplement Note 4: Concentration scale heterogeneity of 210 place types**


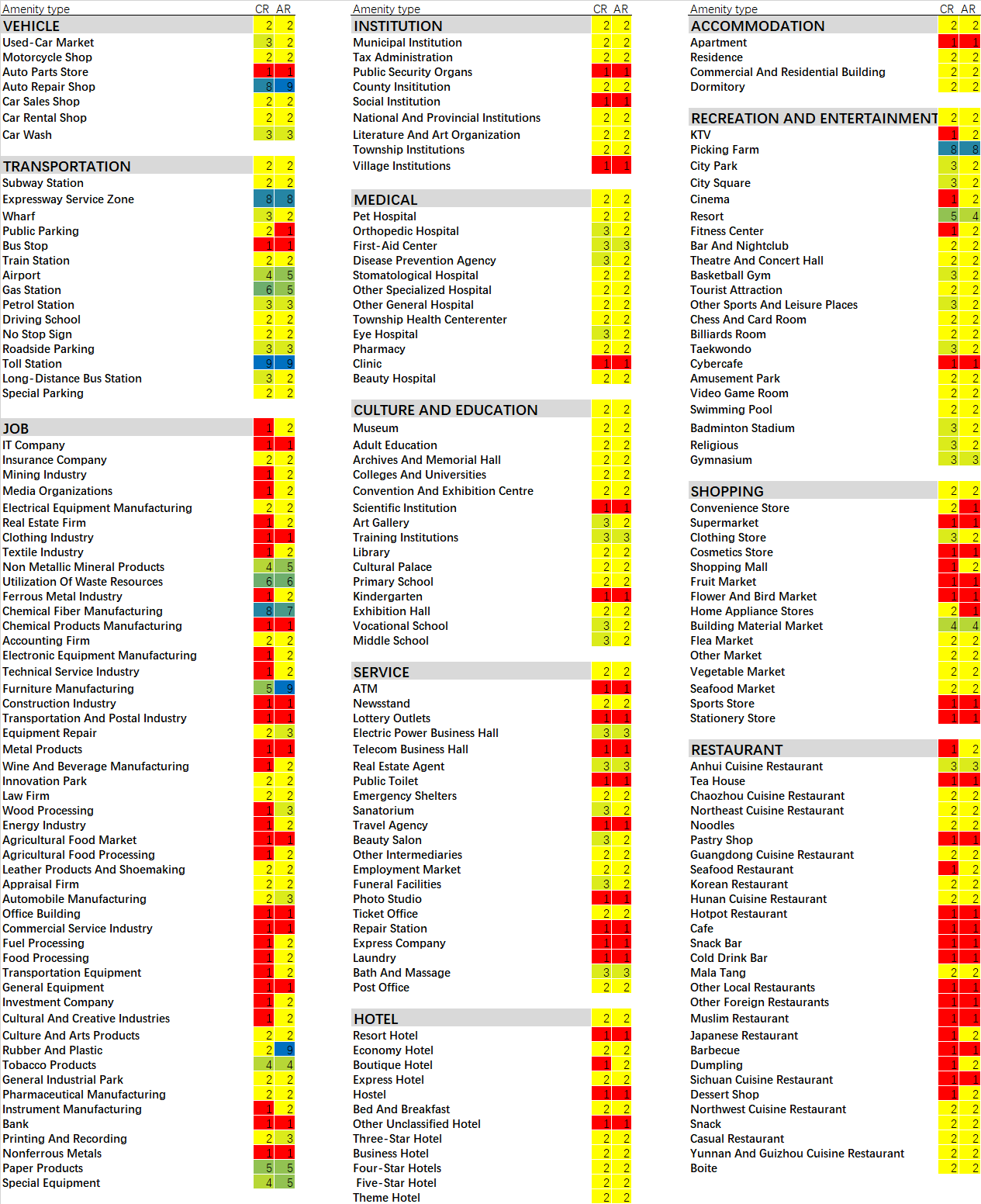


**Supplement Figure 10** The spatial scale (represented by numbers, 1 = 200m, 2 = 400m, 3 = 600m, 4 = 800m, 5 = 1000m, 6 = 2000m, 7 = 3000m, 8 = 4000m, 9 = 5000m) of the maximum Average Weighted Degree for each type of place. CR is short for co-location network, AR is short for adjacent network. The maximum mixing degree for most place types exist at a small scale; For hospital, cultural and educational places it appears at the meso scale; And for job places, the peak mixing value appears either at a large scale (furniture manufacturing, chemical industry, non-metallic minerals and other raw materials or intermediate products manufacturing industries), or a small scale (office, commercial services, cultural and creative industries and other service industries).


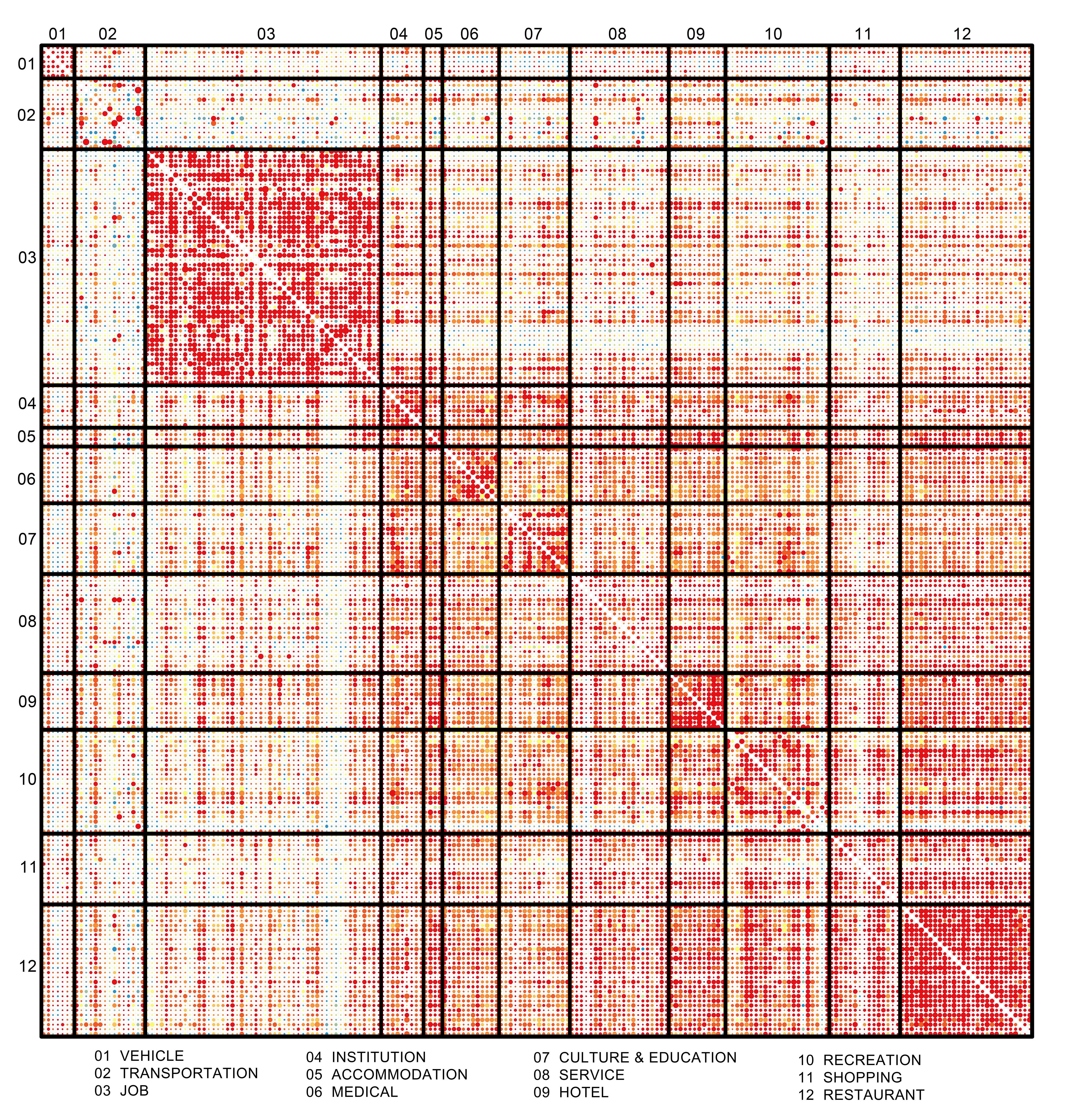


**Supplement Figure 11** The scale at which the maximum value of co-location probability for each place pair appears, red for small scale, blue for large scale, and the size of the point represents the value of the maximum probability for the place pair. The maximum co-location probability of hospital, culture & education and entertainment appears at the meso scale; And for other places the maximum value appears at the small scale. The co-location probability of job places is very high, forming an obvious cluster. Institution, residence, hospital, culture & education, service, hotel, entertainment, restaurant and other types of public places have a high co-location probability between each other, forming another highly mixed cluster.


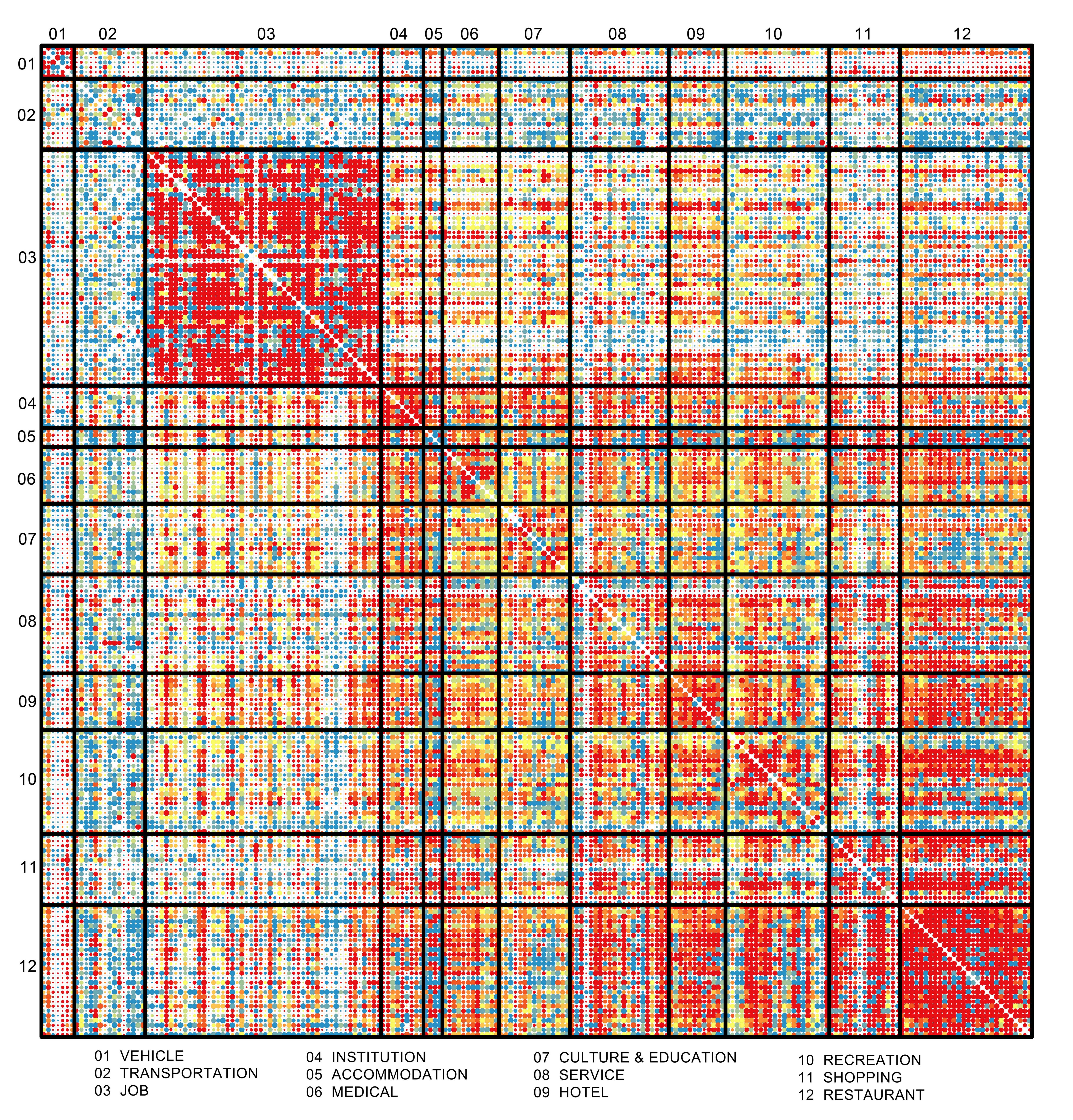


**Supplement Figure 12** The scale at which the highest rank of the co-location probability for each place pair appears among all place pairs, red for small scale, blue for large scale, and the size of the point represents the highest rank for the place pair (the larger point means the higher rank). The highest rank of the place pairs within the category of job, restaurant, hotel and institution appears at the small scale; That within the category of culture & education, hospital, entertainment and institution appears at the meso scale; The highest rank of the place pairs between job and other categories also appears at the meso scale; And that between transportation and other categories appears at the large scale.

**Supplement Note 5: Network modularity of 210 place types**


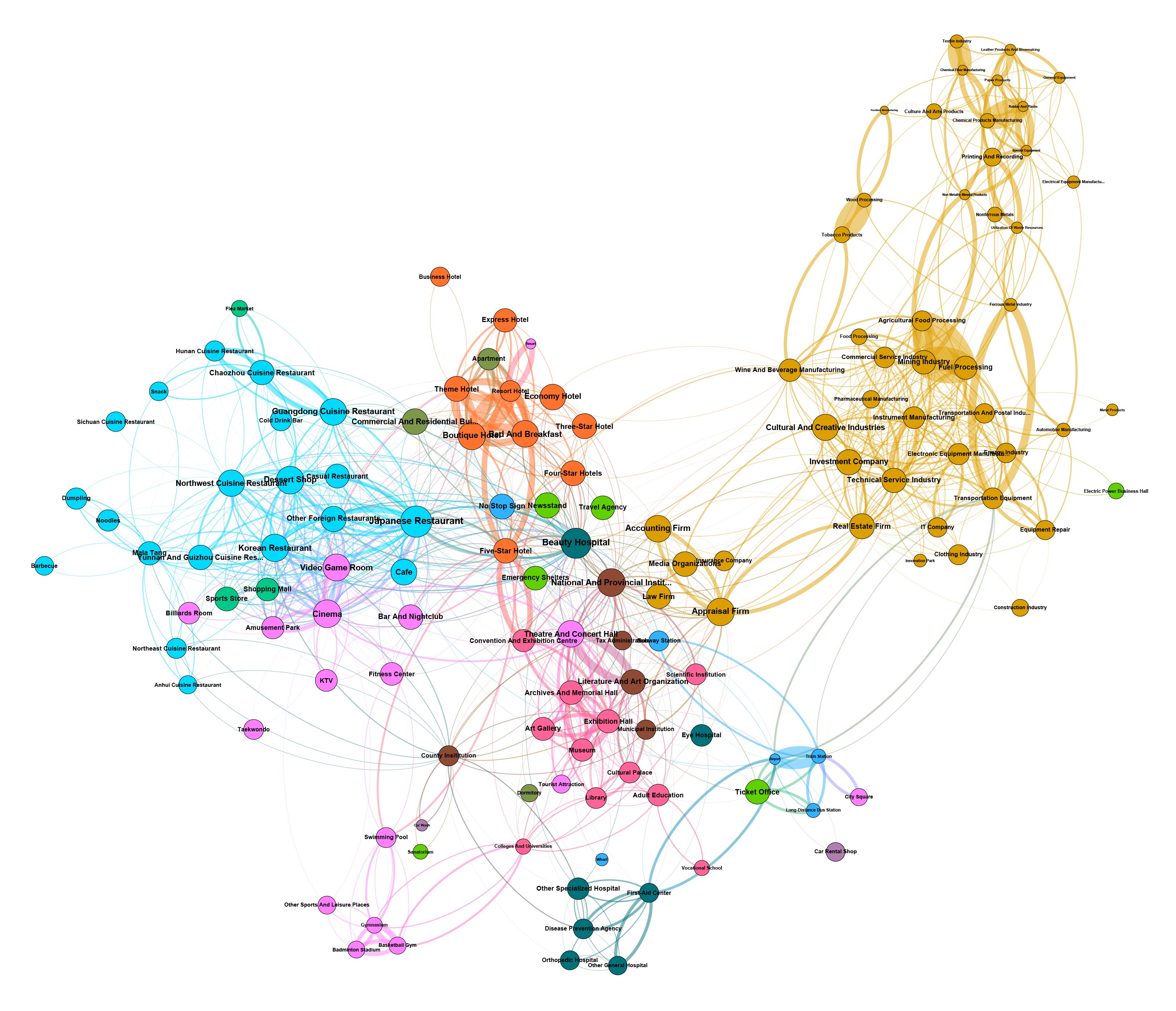


**Supplement Figure 13** Co-location network modularity of 210 place types for all the sample cities at the scale of 200 meters. Several obvious place communities composed of places within the same category can be seen.


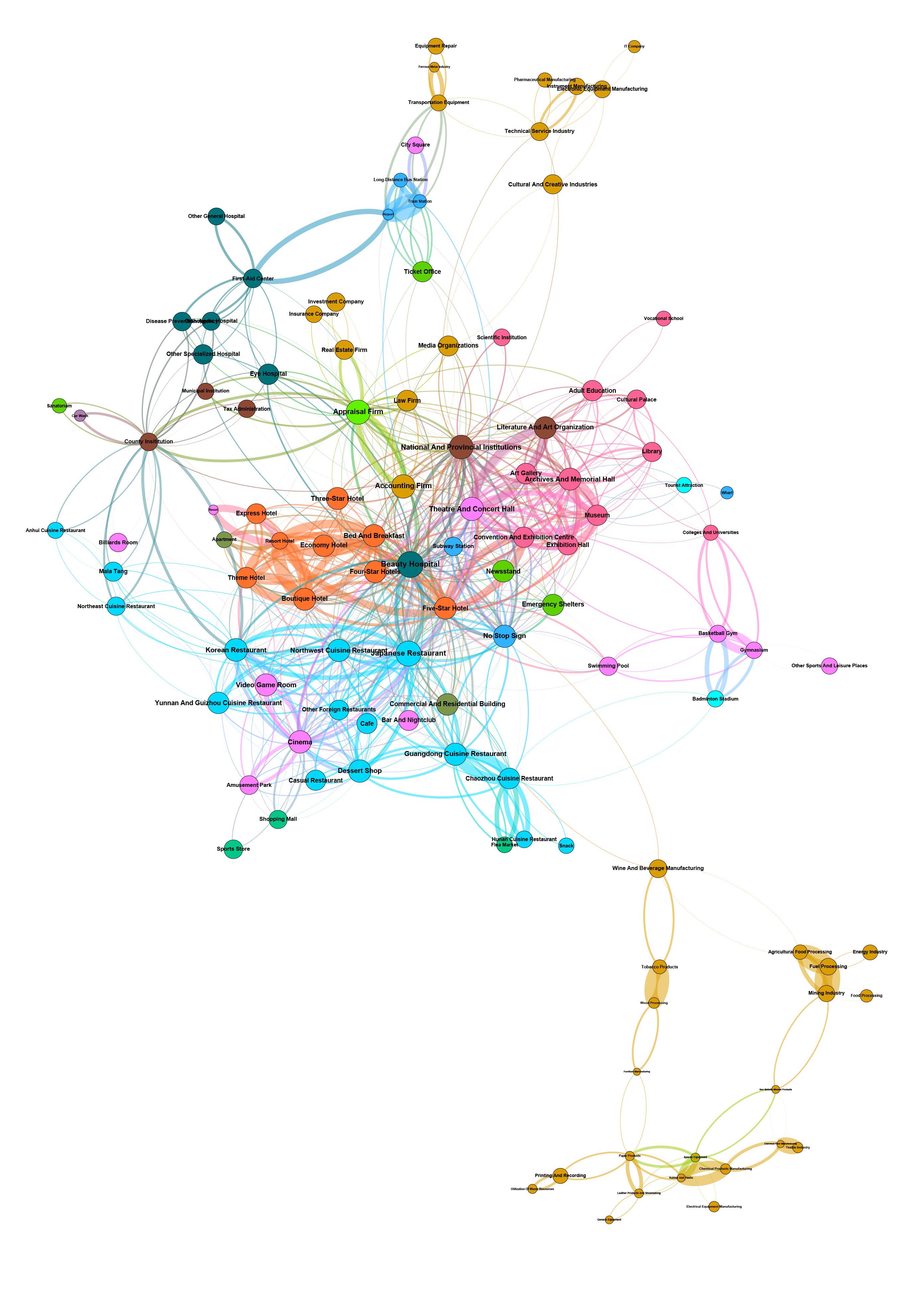


**Supplement Figure 14** Co-location network modularity of 210 place types for all the sample cities at the scale of 400 meters. An obvious place community composed of manufacturing industries can be seen at the bottom right corner.


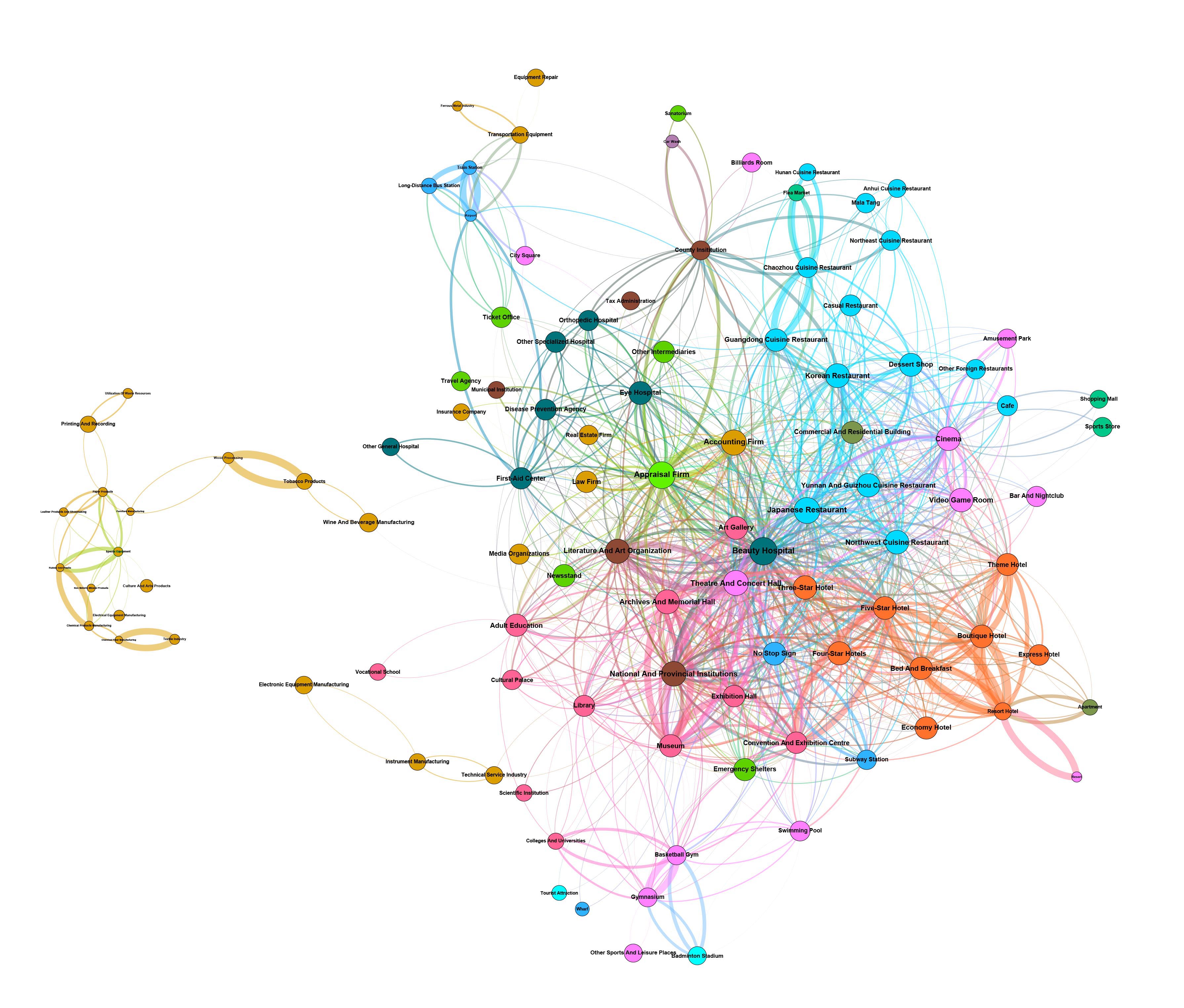


**Supplement Figure 15** Co-location network modularity of 210 place types for all the sample cities at the scale of 600 meters. An obvious place community composed of manufacturing industries can be seen at the left.


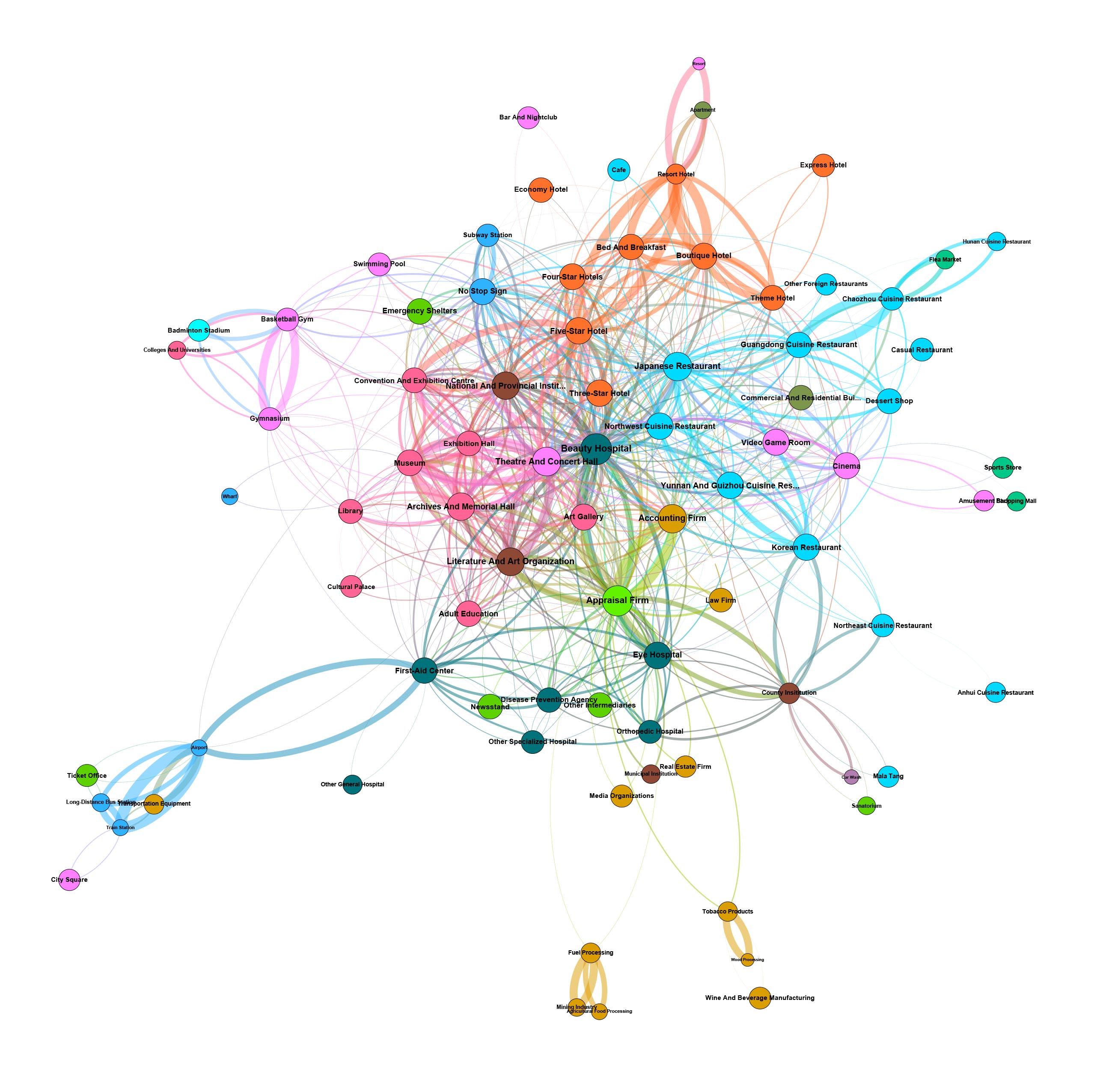


**Supplement Figure 16** Co-location network modularity of 210 place types for all the sample cities at the scale of 800 meters. There are no obvious place communities at this scale.


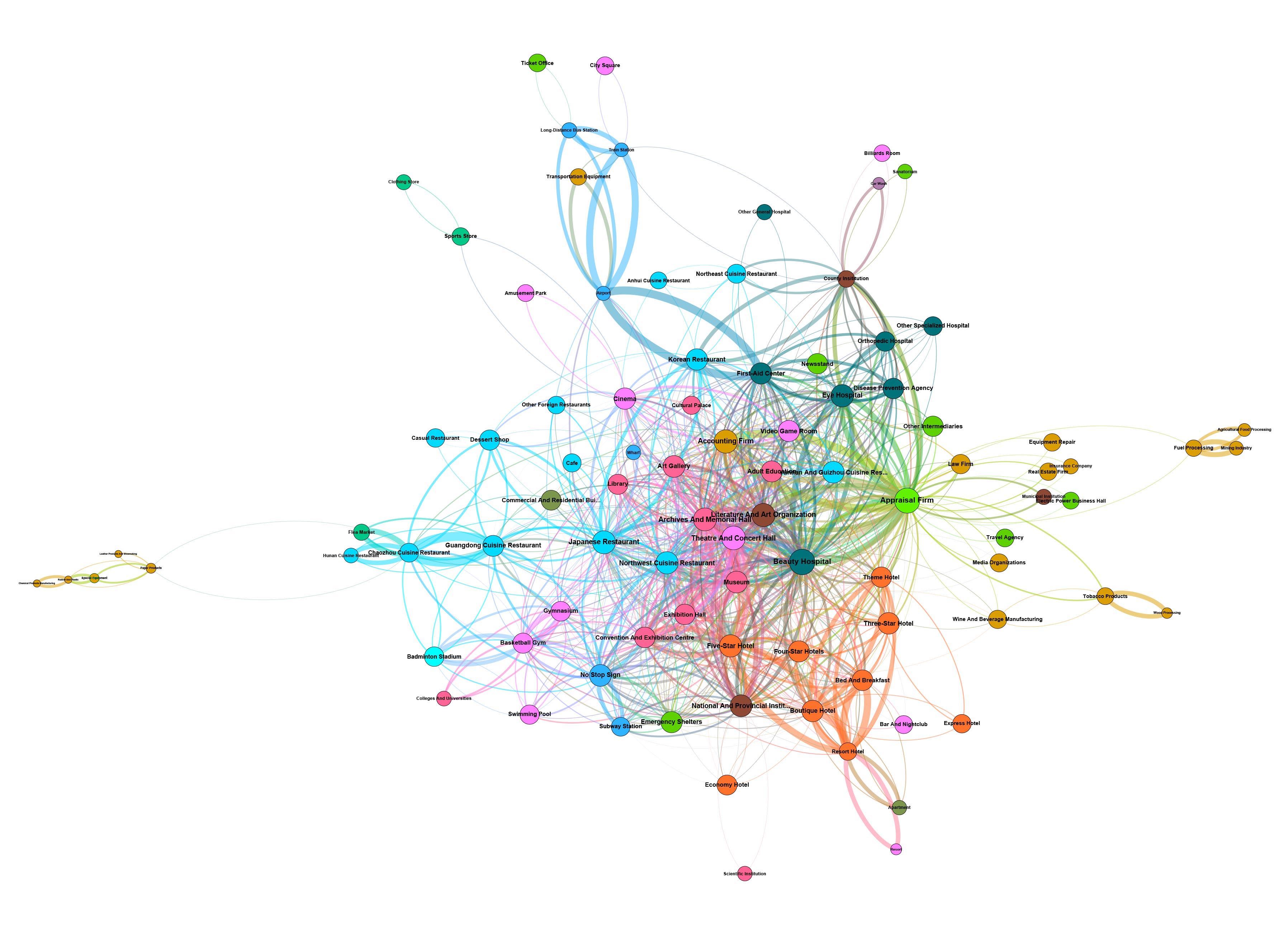


**Supplement Figure 17** Co-location network modularity of 210 place types for all the sample cities at the scale of 1000 meters. There are no obvious place communities at this scale.


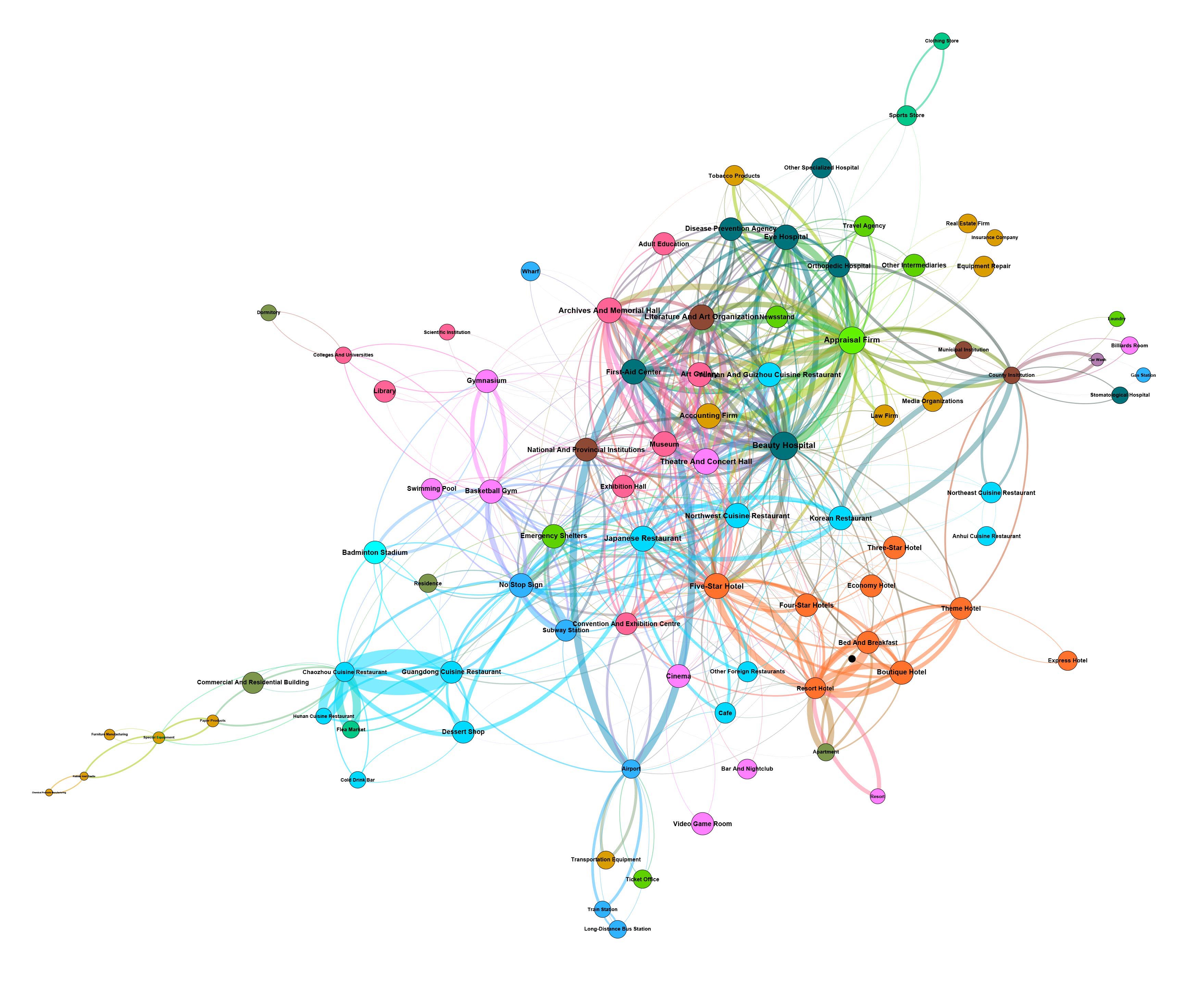


**Supplement Figure 18** Co-location network modularity of 210 place types for all the sample cities at the scale of 2000 meters. There are no obvious place communities at this scale.


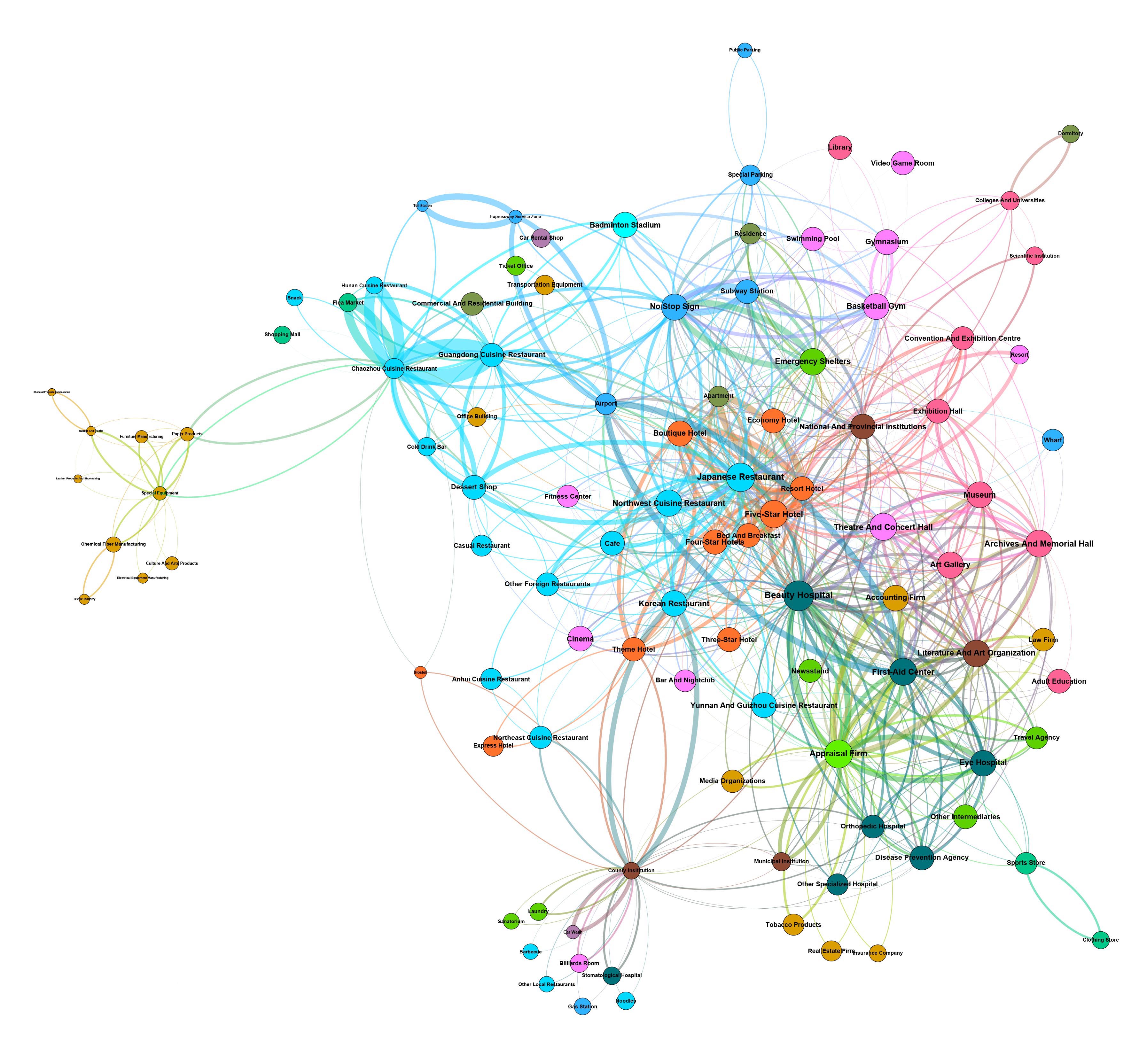


**Supplement Figure 19** Co-location network modularity of 210 place types for all the sample cities at the scale of 3000 meters. A cluster of heavy manufacturing appears at the left.


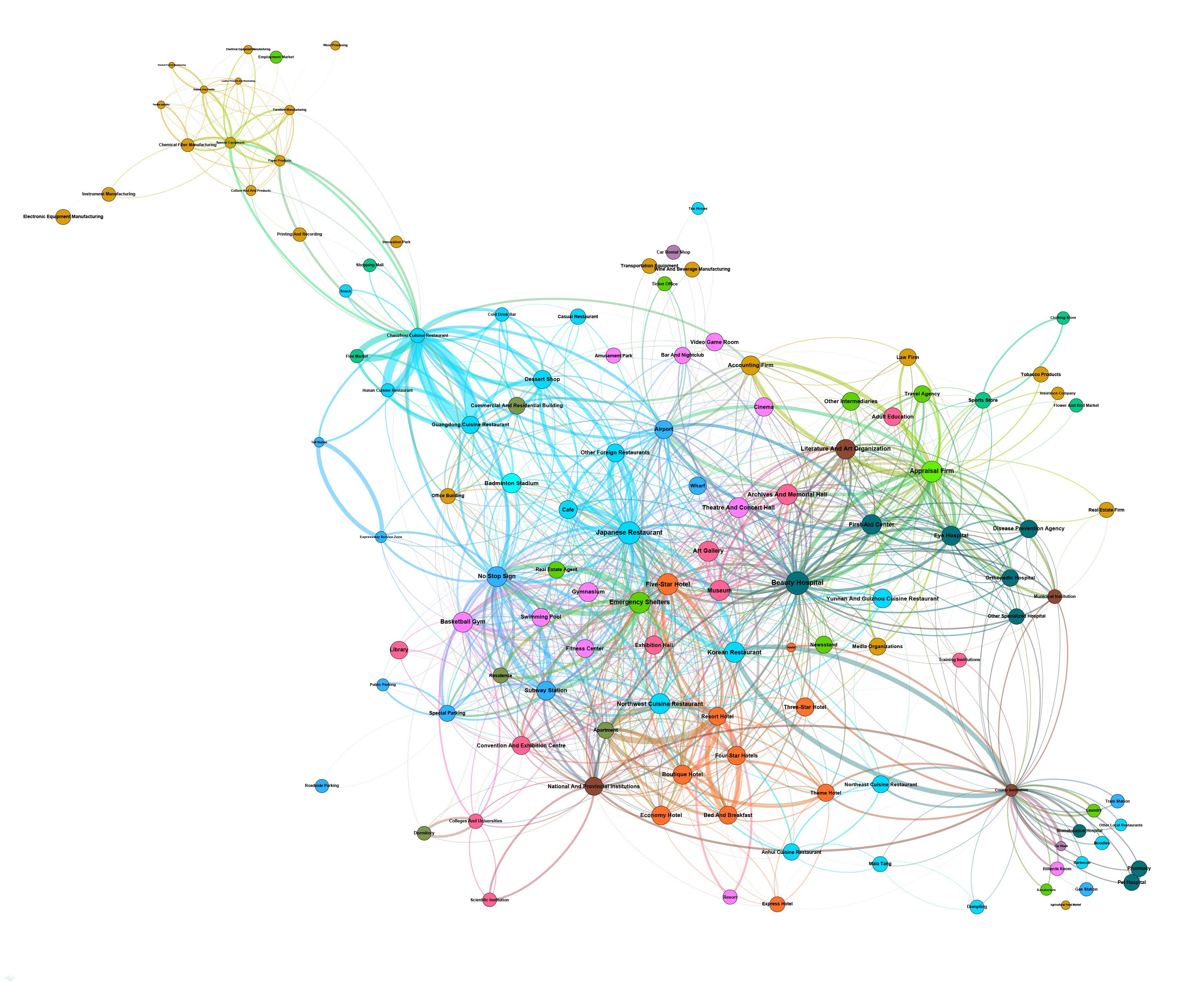


**Supplement Figure 20** Co-location network modularity of 210 place types for all the sample cities at the scale of 4000 meters. A place community of manufacturing industries at the top left corner and another place community centered around county institution at the bottom right corner can be observed.


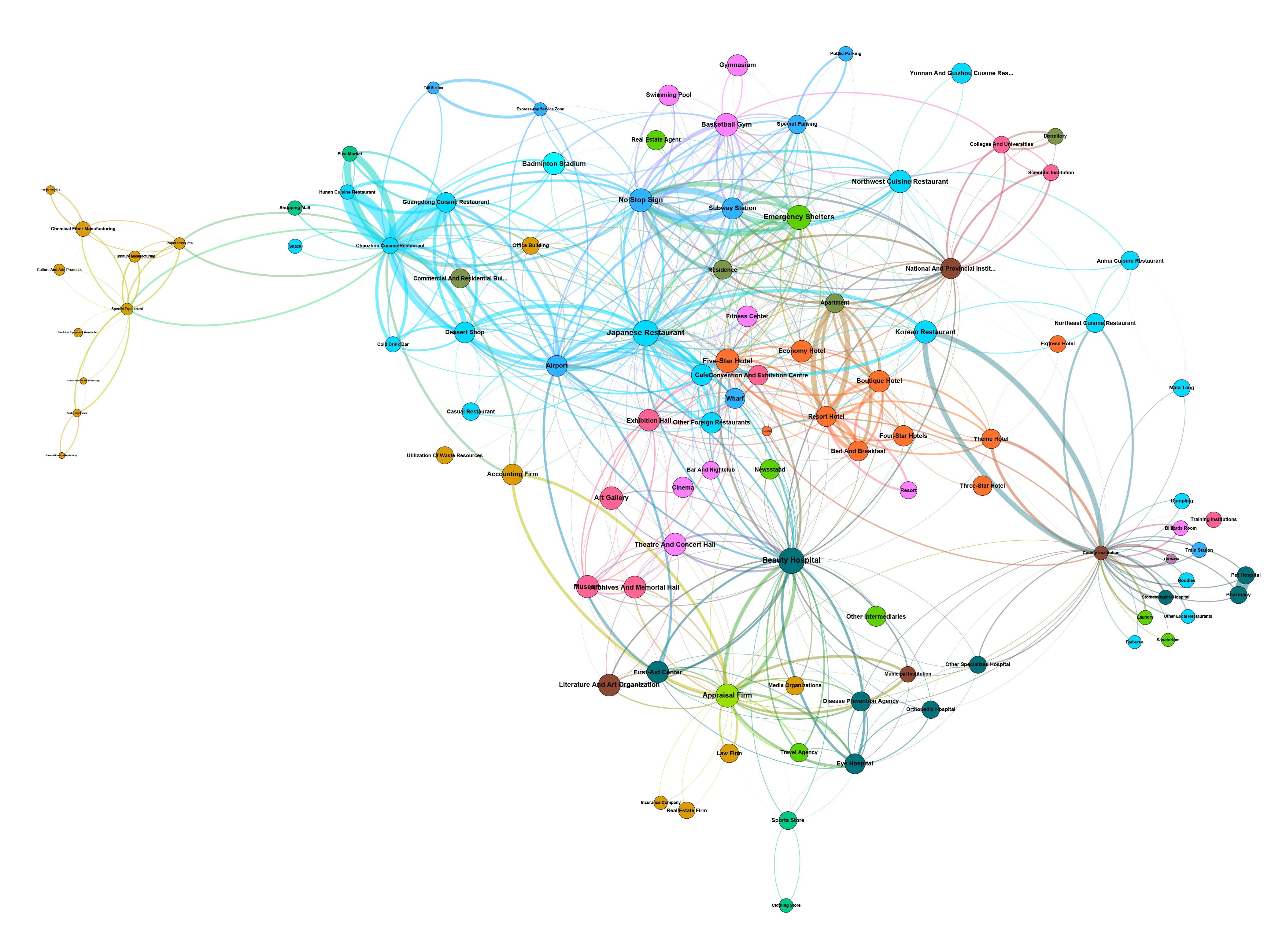


**Supplement Figure 21** Co-location network modularity of 210 place types for all the sample cities at the scale of 5000 meters. Similar to the situation at the scale of 4000 meters, a place community of manufacturing industries at the left and another place community centered around county institution at the bottom right corner can be observed at the scale of 5000 meters.





**Supplement Figure 22** Adjacent network modularity of 210 place types for all the sample cities at all scales. Compared with co-location networks, no obvious place communities can be identified for adjacent networks.


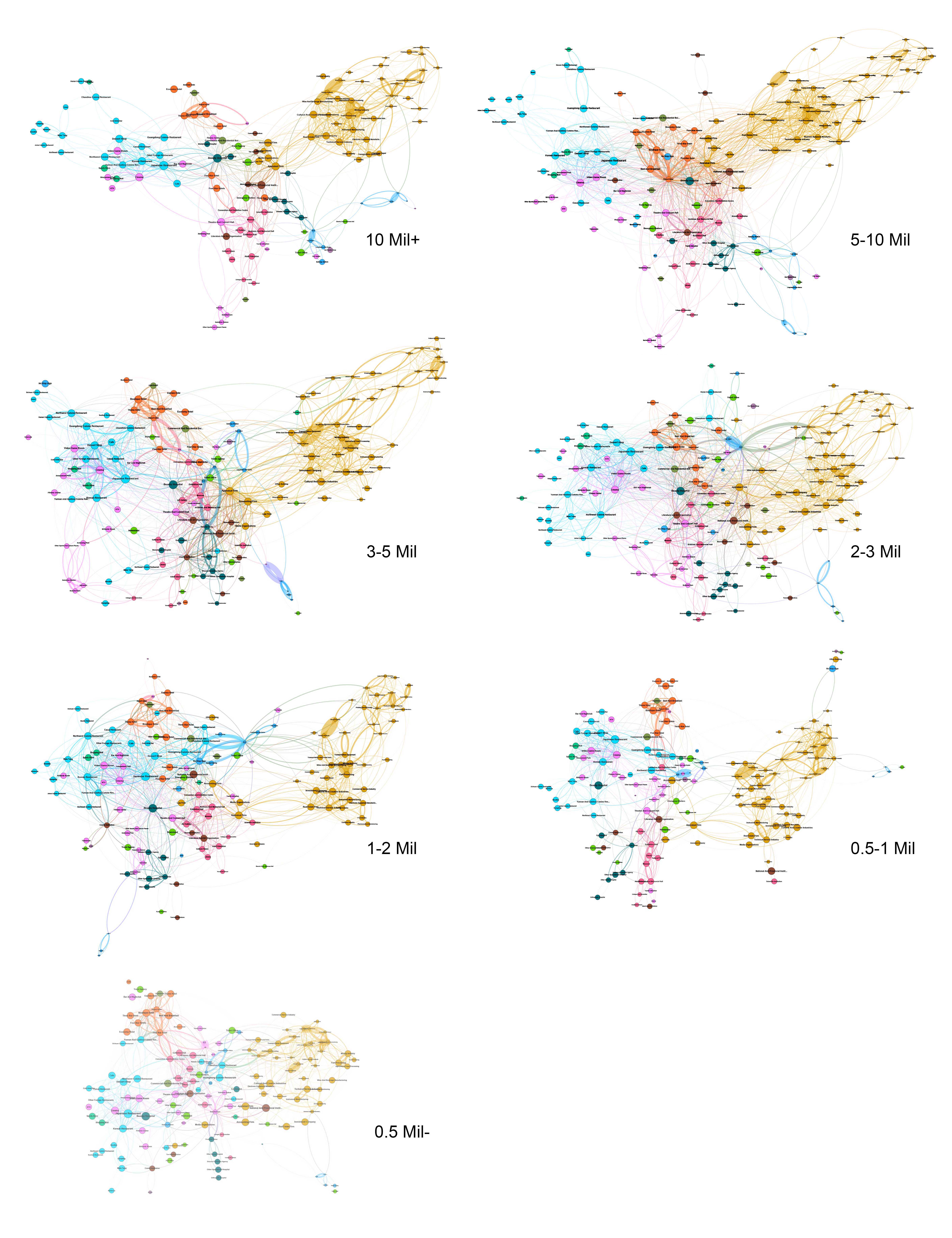


**Supplement Figure 21** Co-location network modularity of 210 place types at the scale of 200 meters for city groups with different urban population. Clustering phenomenon is obvious for city groups with over 3 million population.

**Supplement Note 6: Concentration Scale of Typical Place Clusters**

Due to the limitation of article length, intra-urban place clusters (clustering phenomenon formed by the same place type) are not analyzed in the manuscript. Here we extract typical intra-urban place clusters and discriminate their concentration scales.

When it comes to the study on typical intra-urban place clusters and their spatial scopes, spatial adjacent matrix is used to search for the scale with maximum probability value by comparing the normalized adjacent probability with itself for each type at different scales. To put it simply, six curve types can be summarized by classifying all scale-probability curves of each type of places：1) monotonically increasing curves. 2) monotonically decreasing curves. 3) U-shaped curves with the inflection point to the right. 4) U-shaped curves with the inflection point to the left. 5) inverted U-curves. 6) fluctuating curves.


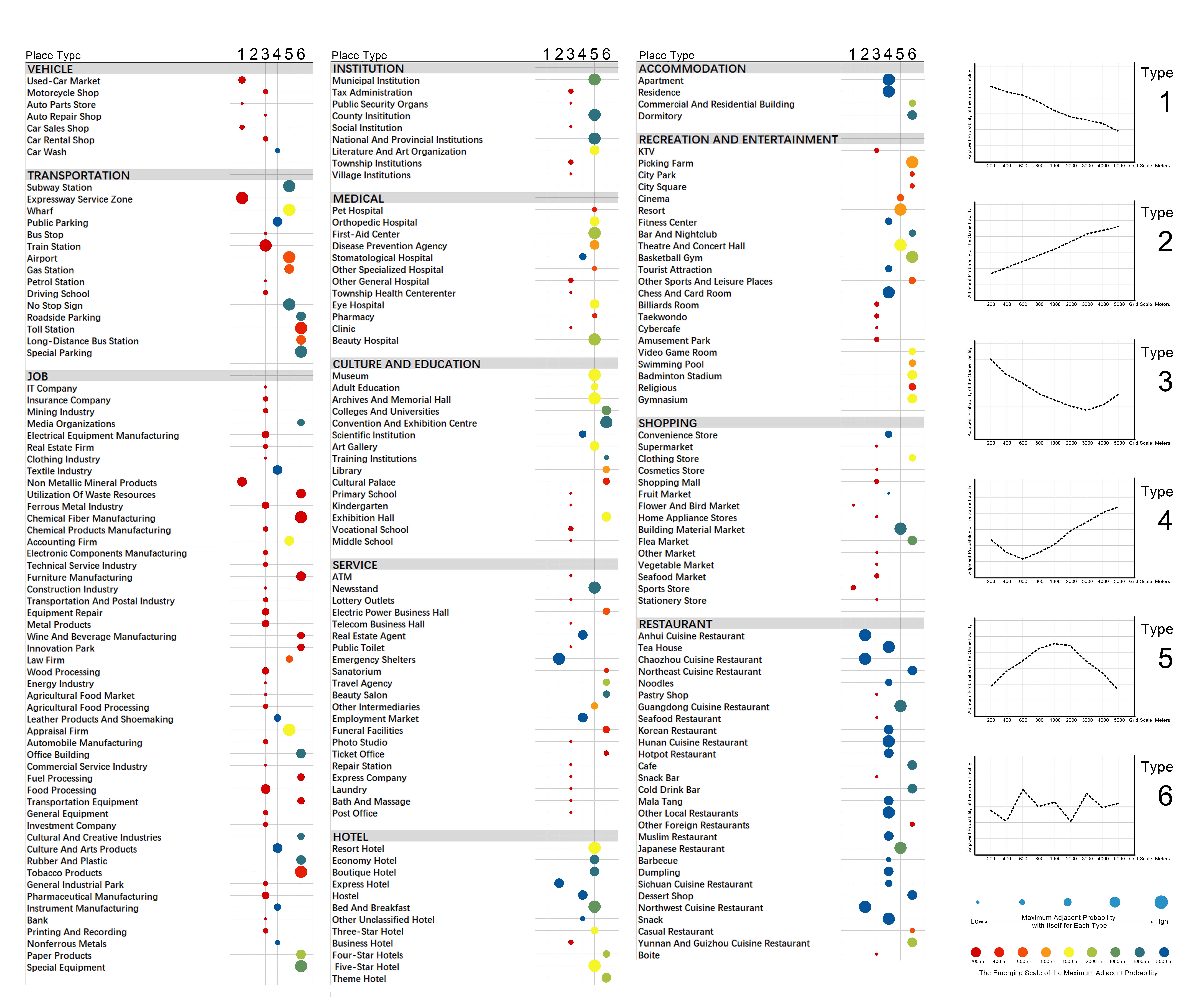


**Supplement Figure 22** The maximum value of the normalized adjacent probability with itself for each type at every scale and its emerging scale for each type of places.

These six curve types can be further be boiled down into three groups.

1) For place clusters in Type 2 and Type 3 which are mainly composed of companies, factories, shops, service facilities and automobile industries, the max value of the normalized adjacent probability usually occurs at the scale of 200-meter-scale or below. It is found that the max values for these clusters are not high as their concentration scales finer than 200 meters cannot be reflected in this study.

2) Place clusters in Type 1 and Type 4, which are mainly composed of restaurants and residences, possess the max value of the normalized adjacent probability occurring at the 5,000-meter-scale or above. The phenomenon of spatial separation between industrial areas and residential & commercial areas is shown by the large max values for these clusters with the scales of 5-10 kilometers.

3) As for place clusters in Type 5, the max value of the normalized adjacent probability usually appears at the scale of 1-2 kilometers. The clusters, which are mainly comprised of hospital, school, transportation and hotels, are large in max values. The fact, they occurring at meso scales between 1,000 to 2,000 meters, suggests that relatively well-defined place clusters are formed by these public places.

4) Strictly speaking, there is no significant clustering phenomenon for Type 6, which encompasses sports, heavy manufacture and beverage stores as the probability-scale curves fluctuate greatly. The possible reason is that the spatial distribution for these places varies greatly across large and small cities (such as heavy manufacture usually gathers in specific resource cities and port cities in China). The mystery may be solved by delving into the sample cities based on urban populations.

It should be noted that some places of large coverage such as hospitals, airports and universities may interfere with the above analysis. Take hospital as an example, a large proportion of hospitals in Chinese cities have expanded over time on their original sites, with the result that the individual hospitals boast huge land footprints (e.g., Beijing Union Medical College Hospital covers 1.6 hectares); and universities usually have larger footprints, such as Tsinghua University, whose main campus occupying an area of about 4 km². The above conclusions on the scale of place clusters should be fully taken into consideration together with the spatial footprint of the place itself.
